# CsMYB60 directly and indirectly activates structural genes to promote the biosynthesis of flavonols and proanthocyanidins in cucumber

**DOI:** 10.1038/s41438-020-0327-z

**Published:** 2020-07-01

**Authors:** Jialin Li, Qianqian Luan, Jing Han, Cunjia Zhang, Mengyu Liu, Zhonghai Ren

**Affiliations:** State Key Laboratory of Crop Biology, Shandong Collaborative Innovation Center of Fruit & Vegetable Quality and Efficient Production, Key Laboratory of Biology and Genetic Improvement of Horticultural Crops in Huang-Huai Region, Ministry of Agriculture, College of Horticultural Science and Engineering, Shandong Agricultural University, Tai’an, 271018 Shandong China

**Keywords:** Molecular biology, Genetics

## Abstract

Flavonols and proanthocyanidins (PAs) are the main pigments in the black spines of cucumber (*Cucumis sativus*) fruit, and *CsMYB60* is a key regulator of the biosynthesis of flavonols and PAs. However, in cucumber, the tissue distribution pattern of flavonols and PAs and the mechanism of their biosynthesis regulated by *CsMYB60* remain unclear. In this study, we clarified the tissue-specific distribution of flavonoids and the unique transcriptional regulation of flavonoid biosynthesis in cucumber. CsMYB60 activated *CsFLS* and *CsLAR* by binding to their promoters and directly or indirectly promoted the expression of *CsbHLH42*, *CsMYC1*, *CsWD40*, and *CsTATA-box binding protein*, resulting in the formation of complexes of these four proteins to increase the expression of *Cs4CL* and interact with CsTATA-box binding protein to regulate the expression of *CsCHS*, thereby regulating the biosynthesis of flavonols and PAs in cucumber. Our data provide new insights into the molecular mechanism of flavonoid biosynthesis, which will facilitate molecular breeding to improve fruit quality in cucumber.

## Introduction

Flavonols and proanthocyanidins (PAs), which are polyphenolic compounds synthesized through the flavonoid biosynthetic pathway, are widely distributed in plants and have a variety of biological functions, including UV light protection, pigmentation, and anti-herbivore and phytopathogen resistance activities^1–6^, in addition to presenting many benefits for human health^7–9^. Flavonols play pivotal roles in modulating reactive oxygen species induced by abscisic acid to control the stomatal aperture^10^ and protect plants against UV light^11,12^. More importantly, flavonols significantly contribute to protection against cardiovascular diseases because of their well-recognized antioxidant, anti-inflammatory, and vasorelaxant actions^13^. PAs play roles in the coloration of plant seeds and in plant adaptation to biotic and abiotic stresses^2,14,15^. Moreover, PAs and their monomeric building blocks (catechin and epicatechin) can act as potential antioxidants showing beneficial effects on human health by protecting against free radical-mediated injury and cardiovascular diseases^7,16^. In addition, PAs also contribute to the taste of multiple beverages and fruits, for instance, tea, wine, and fruit juices^17,18^. Therefore, it is of great importance to clarify the function of flavonols and PAs and the molecular mechanism of their biosynthesis to improve plant stress resistance and fruit quality in cucumber (*Cucumis sativus*).

During plant development, the accumulation of flavonols and PAs is restricted to specific cell types and organs. In white clover (*Trifolium repens*), PAs and their flavan-3-ol precursors were specifically stained with dimethylaminocinnamaldehyde (DMACA) reagent and localized to the epidermal cell layer of the floral organs, showing an adaxial–abaxial asymmetric pattern^19^. PA accumulation is also frequently found specifically in the trichomes on plant organs at relatively low levels^20^. In *Arabidopsis thaliana*, PAs are synthesized and accumulate only in the seed coat and localize to endothelial cells derived from the inner integument^21^, whereas flavonols are found in all tissues^22^. Flavonols accumulate mainly in the skins and flowers of grape berries and in the leaves and stems. No detectable amounts of flavonols have been identified in the seeds and pulp of Shiraz berries and Chardonnay^23,24^. In cucumber, flavonols and PAs are the main pigments of the black spines^6^, but their distribution pattern in other tissues is unknown.

The biosynthetic pathways of flavonols and PAs have been well clarified by studying pigment-deficient mutants of model species^25,26^. In addition to structural genes, many transcription factors (TFs, which act as modulators of gene expression at the transcriptional level through sequence-specific DNA binding or protein–protein interactions during chromatin remodeling) are required for flavonol and PA biosynthesis^27,28^. The key enzyme 4-coumarate: CoA ligase (4CL) provides the precursors for lignin and flavonoid biosynthesis^29^. The first committed step in flavonoid biosynthesis, which is a major pathway of plant secondary metabolism, is catalyzed by chalcone synthase (CHS)^30^. Flavonol synthase (FLS) catalyzes the synthesis of flavonols from dihydroflavonols^31^. An alternative PA monomer (catechin) is thought to be produced from leucoanthocyanidin by leucoanthocyanidin reductase (LAR). *LAR* genes have been isolated from several plant species, and the activity associated with PA accumulation has been characterized^32–34^. In almost all plant species studied so far, the structural genes of the flavonoid biosynthetic pathway are mainly regulated by certain TFs or by a MYB-bHLH-WD40 (MBW) complex at the transcriptional level^35–38^. In *Arabidopsis*, AtMYB11, AtMYB12, and AtMYB111 regulate *AtFLS1* and early steps in the control of flavonol biosynthesis, while an MBW complex activates later flavonoid biosynthetic genes to control the production of anthocyanins and PAs^22,30,39^. MBW regulatory complexes also control PA biosynthesis in strawberry fruits^37^. MtPAR is an MYB transcription factor that has a positive regulatory effect on PA biosynthesis in *Medicago truncatula*^40^. In cucumber, an R2R3-MYB gene, *CsMYB60* was mapped as the best candidate for the *B* (*black spine*) locus in a 50 kb region on chromosome 4^41,42^. Although *CsMYB60* is a known key regulatory gene in the biosynthesis of flavonols and PAs responsible for the coloration of black spines^6^, it is still unclear how *CsMYB60* regulates the structural genes of the flavonol and PA biosynthetic pathways.

Here, we clarified the tissue-specific distribution of flavonols and PAs and the unique mechanism of flavonol and PA biosynthesis regulated by CsMYB60 in cucumber. CsMYB60 was shown to directly activate *CsFLS* and *CsLAR*, two key genes for flavonol and PA biosynthesis, respectively. Additionally, CsMYB60 activated *Cs4CL* transcription through a complex composed of CsMYB60 and its interacting proteins CsbHLH42 (CsaV3_6G037080.1) and/or CsMYC1 (CsaV3_6G000530.1), CsWD40 (CsaV3_1G031140.1), and CsTATA-box binding protein (CsaV3_1G041520.1) and simultaneously promoted the expression of *CsCHS* by interacting with CsTATA-box binding protein, thereby regulating the biosynthesis of flavonols and PAs in cucumber.

## Results

### Distribution patterns of flavonols and PAs in white-spined and black-spined cucumbers

**Fig. 1:**
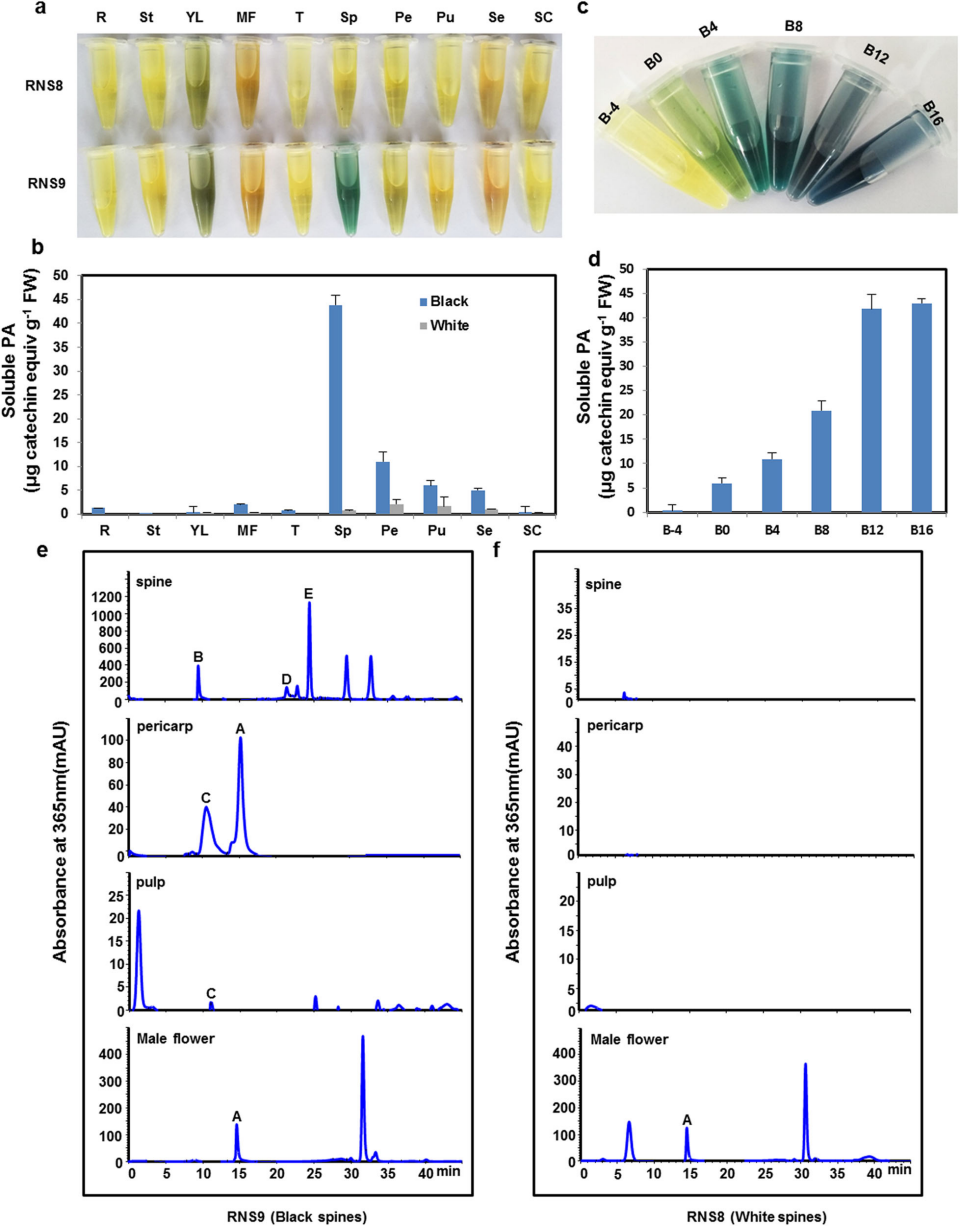
Distribution patterns of flavonols and PAs in the RNS9 (black spines) and RNS8 (white spines) cucumber lines. **a** DMACA (dimethylaminocinnamaldehyde) staining of black-spined and white-spined cucumber lines. **b** Quantification of soluble PAs from (**a**) based on spectrophotometric absorbance. **c** DMACA staining of cucumber black spines at different developmental stages. **d** Quantification of soluble PAs from (**c**) based on spectrophotometric absorbance. **e**, **f** HPLC analysis of flavonols in different tissues of black-spined and white-spined cucumber lines. Absorbance was monitored at 365 nm. Peaks A–E represent five kinds of flavonols: quercetin-3-O-rutinoside-7-O-glucose (A), kaempferol-3-O-rutinoside-7-O-glucoside (B), isorhamnetin-3-O-rutinoside-7-O-glucoside (C), kaempferol-3-O-rutinoside (D), and isorhamnetin-3-O-rutinoside (E). R, root; St, stem; YL, young leaf; MF, male flower; Sp, spine at 12 days after anthesis (DAA); T, tendril; Pe, pericarp; Pu, pulp; Se, seed; SC, seed coat. B-4, black spine at 4 days before anthesis (DBA); B0, black spine at 0 DBA; B4, black spine at 4 DAA; B8, black spine at 8 DAA; B12, black spine 12 at DAA; B16, black spine at 16 DAA

The main pigments of the black spines of cucumber have been identified as flavonols and PAs^6^. Hence, we sought to determine the patterns and contents of flavonols and PAs in different tissues between white-spined and black-spined cucumber lines. Therefore, we performed DMACA staining for the analysis of PA contents in different tissues. The results indicated that large amounts of PAs specifically accumulated in black spines, and there were also detectable amounts in the pericarp, pulp, seed coat, and male flowers of the black-spined cucumber line, while there were detectable amounts of PAs only in the pericarp, pulp, and seed coat of the white-spined line (Fig. 1a, b). Catechin contents of approximately 43.8, 10.9, 5.9, 5.0, 2.0, and 1.2 μg per gram fresh weight (gFW) were detected in the spines, pericarp, pulp, seed coat, male flowers, and roots, respectively, of the black-spined cucumber, while the contents in the white-spined cucumber were only 2.0, 1.5, and 1.0 μg/gFW in the pericarp, pulp, and seed coat, respectively (Fig. 1b).

The coloration of black spines occurs gradually and is complete at 12 days after anthesis (DAA); the accumulation of flavonols is consistent with the coloration process of black spines^6^. Here, our data showed that PA accumulation presented the same trend as the coloration of black spines (Fig. 1c, d). These data further strengthened the conclusion that flavonols and PAs were the main pigments determining the coloration of black spines^6^ (Fig. 1c, d).

In contrast to the PA distribution pattern, flavonols were distributed in all tested tissues of black-spined cucumber, but high levels of flavonols accumulated only in the male flowers and leaves of white-spined cucumber (Figs. 1e, f and S1). Five flavonols were verified: quercetin-3-O-rutinoside-7-O-glucose, kaempferol-3-O-rutinoside-7-O-glucoside, isorhamnetin-3-O-rutinoside-7-O-glucoside, kaempferol-3-O-rutinoside, and isorhamnetin-3-O-rutinoside^6^ (Figs. 1e, f and S1).

### Overexpression of *CsMYB60* increases the accumulation of flavonols and PAs in different tissues, especially in spines

**Fig. 2:**
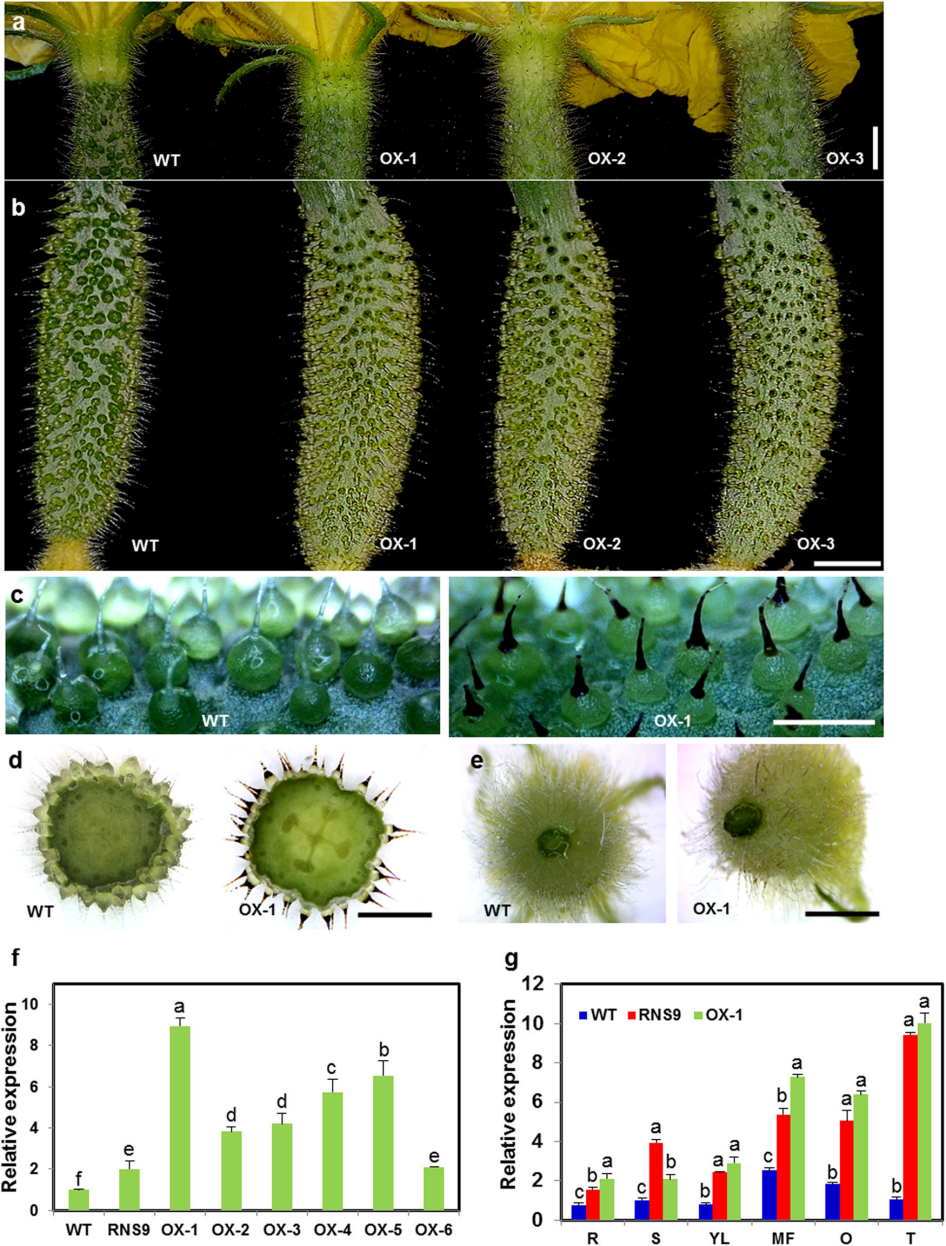
Phenotypic analysis of *35S*:*CsMYB60* transgenic cucumber lines. **a** Cucumber ovaries at 0 DBA. **b** Cucumber fruits at 4 DAA. **c** The spine color of cucumber fruits at 4 DAA from the Xintaimici (wild type, WT) and OX-1 lines observed under a stereoscopic microscope. **d** Cross-section of cucumber fruits at 4 DAA from the WT and OX-1 lines observed under a stereoscopic microscope. **e** The trichome color of male flower receptacles at 0 days before anthesis (DBA) from the WT and OX-1 lines observed under a stereoscopic microscope. **f** Relative expression levels of *CsMYB60* in the WT, RNS9, and *35S*:*CsMYB60* transgenic lines. The young leaves of the WT, RNS9, and *35S*:*CsMYB60* transgenic lines were used for qRT-PCR analysis. **g** Relative expression levels of *CsMYB60* in different tissues from WT, RNS9, and OX-1. R, young root at 10 days after sowing; S, young stem; YL, young leaf; MF, male flower at 0 DBA; O, ovary at 0 DBA; T, young tendril obtained from plants after 8 weeks in the soil. The cucumber *β-actin* gene was used as an internal control for normalization, and three biological replicates were performed for these experiments. Error bars indicate SE. Different letters indicate significant differences (*P* < 0.05). DBA, days before anthesis; DAA, days after anthesis. Scale bars: 0.5 cm in (**a**) and (**b**); 1 mm in (**c**)–(**e**)

As the best candidate gene for the *B* locus^41^, *CsMYB60* has been verified to be a key regulator of the biosynthesis of flavonols and PAs in black spines through a transient expression assay^6^. Moreover, CsMYB60 shares the highest similarity among its homologous proteins with AtMYB111, which functions in flavonoid biosynthesis^30^ (Fig. S2). To obtain additional genetic evidence and more information about the function of *CsMYB60* in the biosynthesis of flavonols and PAs, we constitutively expressed *CsMYB60* in Xintaimici with white fruit spines (wild type, WT), which is a north China-type cucumber inbred line. A total of six independent first-generation transgenic lines (T_1_, OX1-6) were obtained, and *CsMYB60* expression levels were significantly higher in all six T_1_ lines compared with WT and even compared with the black-spined line RNS9 (except for OX6) (Fig. 2f).

Based on the *CsMYB60* expression level and the phenotype of black spines, OX1 was selected from the black-spined transformants for further studies (Figs. 2a, b and S3). OX1 showed a similar process of pigment accumulation in the fruit spines and a similar pattern in the flower trichomes to RNS9 (Figs. 2a–e and S3). Quantitative real-time RT-PCR (qRT-PCR) analysis showed that the *CsMYB60* expression levels were higher in various tested tissues of OX1 than in those of WT, similar to the pattern observed in RNS9 (Fig. 2g).

To verify the identity of the accumulated pigments as flavonols and PAs and their distribution patterns in OX1, T_2_ nontransgenic and transgenic siblings isolated from T_1_ selfing progenies of OX1 were used for the analyses of flavonols and PAs via high-performance liquid chromatography (HPLC) and DMACA staining. The flavonol contents were significantly increased to different extents in all tested tissues of the transgenic siblings, showing similar patterns to those in RNS9 (Figs. 1e, S1, S4a, b, and S5); similarity to RNS9 was also observed for the PA contents, which were significantly increased in most tested tissues of the transgenic siblings compared to nontransgenic siblings (Figs. 1b and S4c). Moreover, we examined the expression of key genes encoding enzymes responsible for flavonoid biosynthesis by real-time PCR analysis in the transgenic and nontransgenic lines. *CsMYB60* and five structural genes, *Cs4CL*, *CsCHS*, *CsFLS*, *CsDFR*, and *CsLAR*, were significantly upregulated in the transgenic siblings (Fig. S4d).

### CsMYB60 directly activates *CsFLS* and *CsLAR* by binding to their promoters

**Fig. 3:**
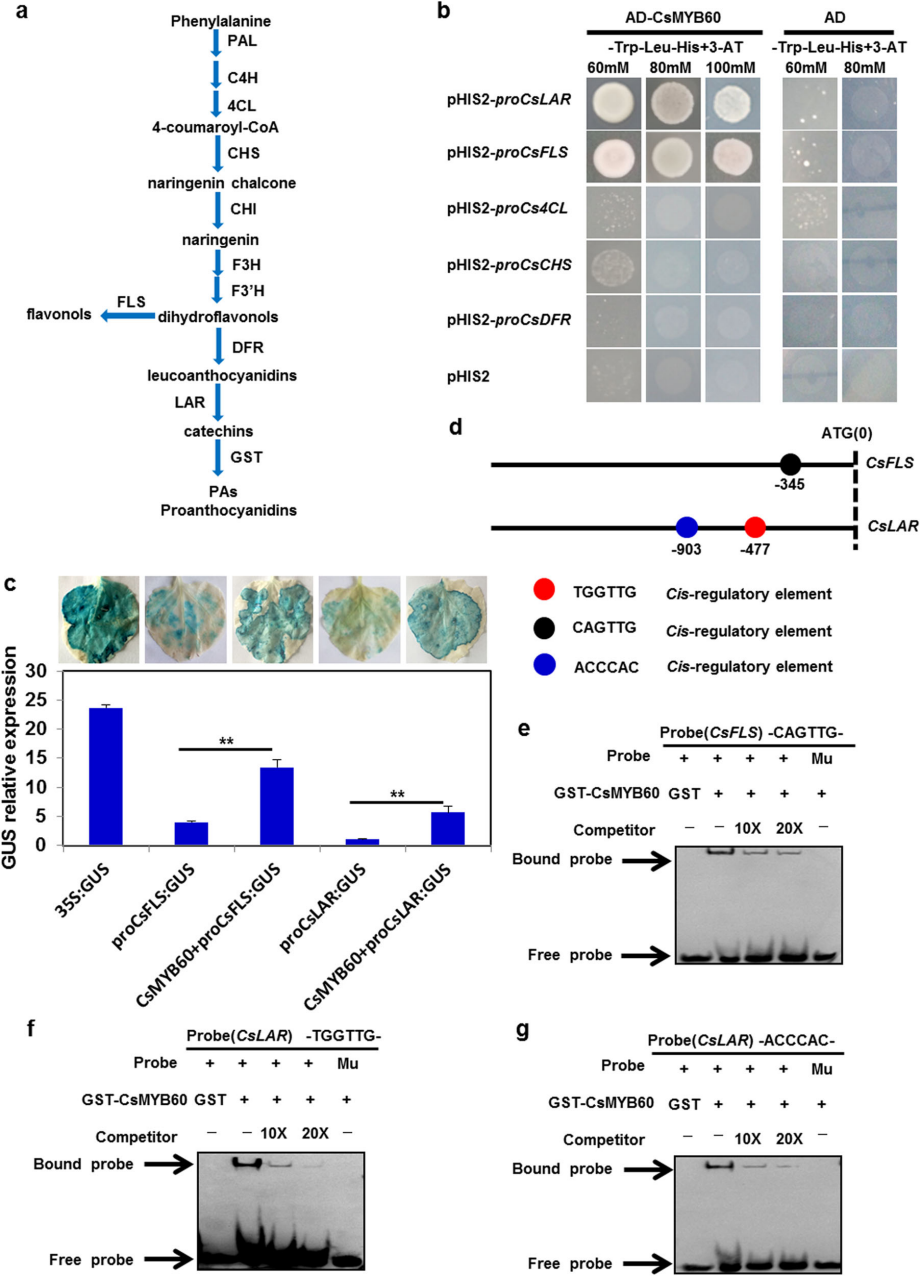
CsMYB60 directly bound to the promoters of *CsFLS* and *CsLAR*. **a** Simplified diagram of the flavonoid biosynthetic pathway. CHS, chalcone synthase; CHI, chalcone isomerase; C4H cinnamate 4-hydroxylase; 4CL, 4-coumaroyl CoA ligase; DFR, dihydroflavanol 4-reductase; F3H, flavanone 3-hydroxylase; F3′H, flavonoid 3′-hydroxylase; FLS, flavonol synthase; PAL, phenylalanine ammonia lyase; GST, glutathione S-transferase; LAR, leucoanthocyanidin reductase. **b** CsMYB60 could bind to the promoters of *CsFLS* and *CsLAR* in a yeast one-hybrid assay. **c** CsMYB60 activated the expression of *GUS* driven by *CsFLS* and *CsLAR*, respectively, in transient tobacco leaf assays. Three biological replicates were performed for the gene expression analyses. Error bars represent SE. Statistical significance: *P < 0.05, **P < 0.01. **d** Schematic diagram of CsMYB60 binding *cis*-elements in the promoters of *CsFLS* and *CsLAR*. The translational start site (ATG) is shown at position 0. **e**–**g** GST-CsMYB60 could directly bind to the motifs of the *CsFLS* and *CsLAR* promoters in EMSAs. The GST protein was incubated with the labeled probe in the first lane to serve as a negative control. 10- and 20-fold excesses of unlabeled probes were added for competition. The mutant probe contained two nucleotide mutation

Since CsMYB60 promotes the expression of *Cs4CL*, *CsCHS*, *CsFLS*, *CsDFR*, and *CsLAR*, which are the key genes in flavonoid biosynthesis (Figs. 3a and S4d), we wanted to know how *CsMYB60* regulates these genes. Therefore, yeast one-hybrid (Y1H) assays were performed to determine whether CsMYB60 binds to the promoters of these genes. The Y1H results showed that CsMYB60 could directly bind to the promoters of *CsFLS* and *CsLAR*, but not the promoters of *Cs4CL*, *CsCHS*, and *CsDFR* (Fig. 3b). Transient expression assays with the β-glucuronidase reporter gene (*GUS*) in the leaves of tobacco (*Nicotiana benthamiana*) showed that CsMYB60 could activate the expression of *CsFLS* and *CsLAR in vivo* (Fig. 3c).

To identify the *cis*-elements to which CsMYB60 could bind, different fragments from the *CsFLS* and *CsLAR* promoters were tested in Y1H assays. We identified one *cis*-element (CAGTTG) in the *CsFLS* promoter, which belonged to the MYB-core type I element^43,44^, and two *cis*-elements (TGGTTG and ACCCAC) in the *CsLAR* promoter, which were a MYB-core type II element^43^ and an AC-rich element^22^ (Figs. 3d and S6). These three *cis*-elements were further proven to be bound by CsMYB60 through EMSAs (Fig. 3e–g).

### CsMYB60 physically interacts with CsbHLH42, CsMYC1, CsWD40, and CsTATA-box binding protein and directly or indirectly activates their expression

**Fig. 4:**
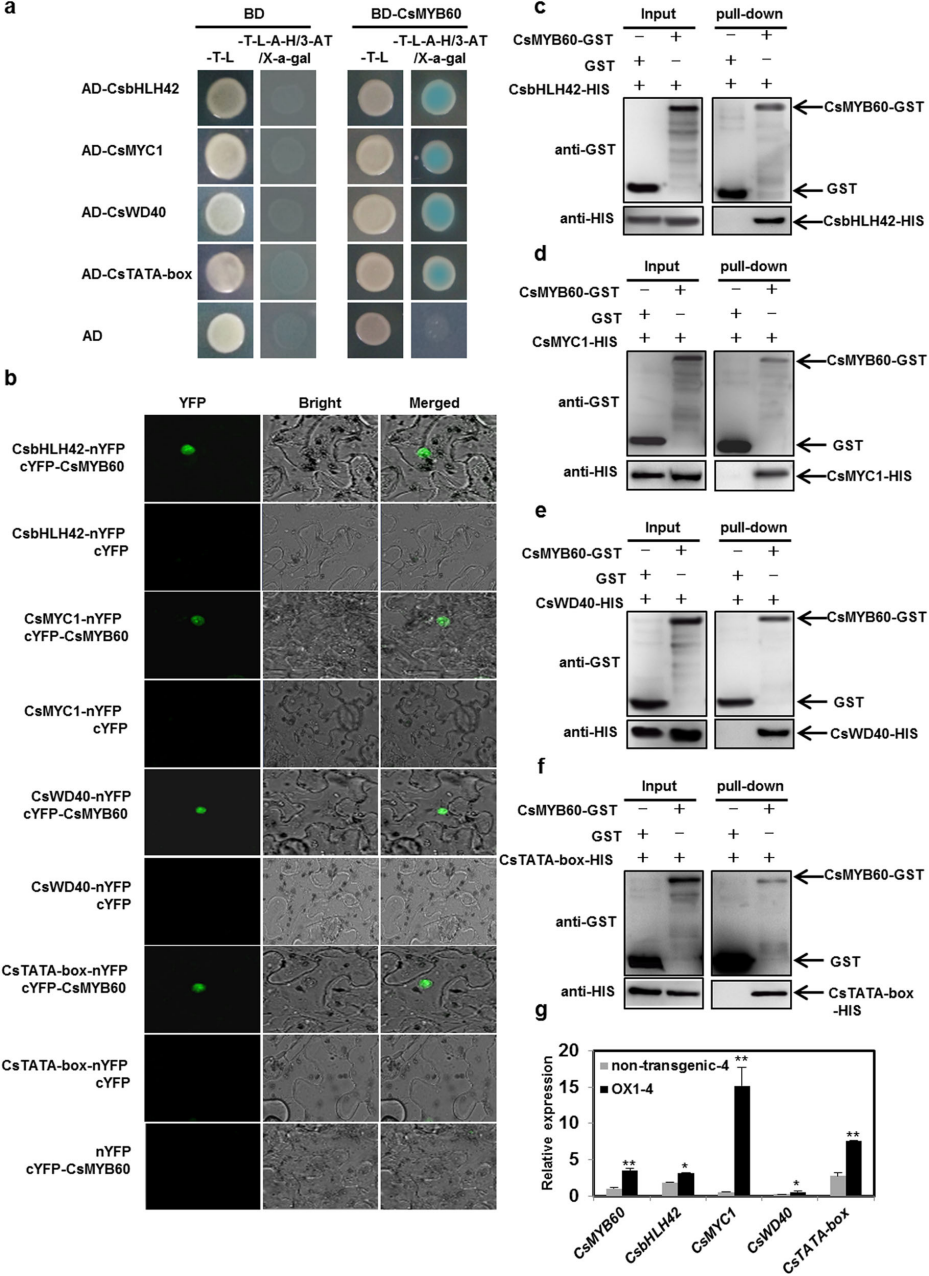
CsMYB60 physically interacted with CsbHLH42, CsMYC1, CsWD40, and CsTATA-box binding protein. **a** Yeast two-hybrid assays. **b** Bimolecular fluorescence complementation (BiFC) assays. **c**–**f** Pull-down assays validating the interaction of CsMYB60 with CsbHLH42 (**c**), CsMYC1 (**d**), CsWD40 (**e**), and CsTATA-box binding protein (**f**). **g** Relative expression levels of *CsMYB60*, *CsbHLH42*, *CsMYC1*, *CsWD40*, and *CsTATA-box binding protein* in spines at 4 days before anthesis in OX-1 and its nontransgenic siblings. The cucumber *β-actin* gene was used as an internal control. Three biological replicates were performed for the gene expression analyses. Error bars represent standard errors (SE). Statistical significance: *P < 0.05, **P < 0.01

CsMYB60 greatly induced the expression of *Cs4CL* and *CsCHS* (Fig. S4d) but could not bind to their promoters (Fig. 3b). To search for CsMYB60-interacting partners that could bind to the *Cs4CL* and *CsCHS* promoters, we employed CsMYB60 as bait to screen a cucumber cDNA library via yeast two hybrid (Y2H) assays, and 12 putative interacting proteins were obtained (Table 1). Then, the interactions between these 12 proteins and CsMYB60 were confirmed via Y2H assays (Figs. 4a and S7). Among the 12 putative interacting proteins, we focused on the four transcription factors CsbHLH42 (CsaV3_6G037080.1), CsMYC1 (CsaV3_6G000530.1), CsWD40 (CsaV3_1G031140.1), and CsTATA-box binding protein (CsaV3_1G041520.1), which were repeatedly identified when we screened the Y2H library. Then, we confirmed the interactions of CsMYB60 with CsbHLH42, CsMYC1, CsWD40, and CsTATA-box binding protein in bimolecular fluorescence complementation (BiFC) and pull-down assays (Fig. 4b–f).

**Table 1:** Proteins screened in yeast two hybrid assays

Protein ID	Protein function annotation	Protein ID	Protein function annotation
*CsaV3_1G031140.1*	WD-40 repeat family protein	*CsaV3_3G004070.1*	Transporter
*CsaV3_6G037080.1*	Basic helix-loop-helix (bHLH) family protein	*CsaV3_6G039130.1*	Ubiquitin-protein ligase
*CsaV3_6G000530.1*	Basic helix-loop-helix (bHLH) family protein	*CsaV3_1G041520.1*	TATA-box-binding protein
*CsaV3_1G031230.1*	COL1 (constans-like 1); transcription factor/zinc ion binding	*CsaV3_3G020110.1*	Glyceraldehyde-3-phosphate dehydrogenase
*CsaV3_6G016480.1*	GIL1 (GRAVITROPIC IN THE LIGHT)	*CsaV3_1G046680.1*	PSAF (photosystem I subunit F)
*CsaV3_2G024370.1*	SKP1/ubiquitin-protein ligase	*CsaV3_6G021530.1*	40S ribosomal protein S23

If CsMYB60 interacts with CsbHLH42, CsMYC1, CsWD40, and CsTATA-box binding protein *in vivo*, they must be expressed at the same time and in the same place. To verify this, we analyzed the spatiotemporal expression patterns of these five genes. The qRT-PCR analysis demonstrated that they were all expressed at higher levels in black spines (from RNS9) than in white spines (from RNS8), at least in 4 days before anthesis (Fig. S8). We also found that there were higher expression levels of these five genes in the black spines of the transgenic siblings of OX-1 than in the white spines of the nontransgenic siblings of OX-1 at 4 days before anthesis, even though their expression levels showed large differences (Fig. 4g).

The data indicated that *CsMYB60* was expressed earlier than the *CsMYC1*, *CsbHLH42*, *CsTATA-box*, and *CsWD40* genes (Fig. S8), and these four genes were all upregulated in the transgenic lines overexpressing *CsMYB60* (Fig. 4g). Therefore, we speculated that CsMYB60 might regulate their expression. Using Y1H assays, we found that CsMYB60 could directly bind to the promoters of *CsbHLH42* and *CsTATA-box binding protein* but not the promoters of *CsMYC1* and *CsWD40* (Fig. S9).

### CsMYB60 can form complexes with its interaction partners to activate the expression of *Cs4CL* and *CsCHS*

**Fig. 5:**
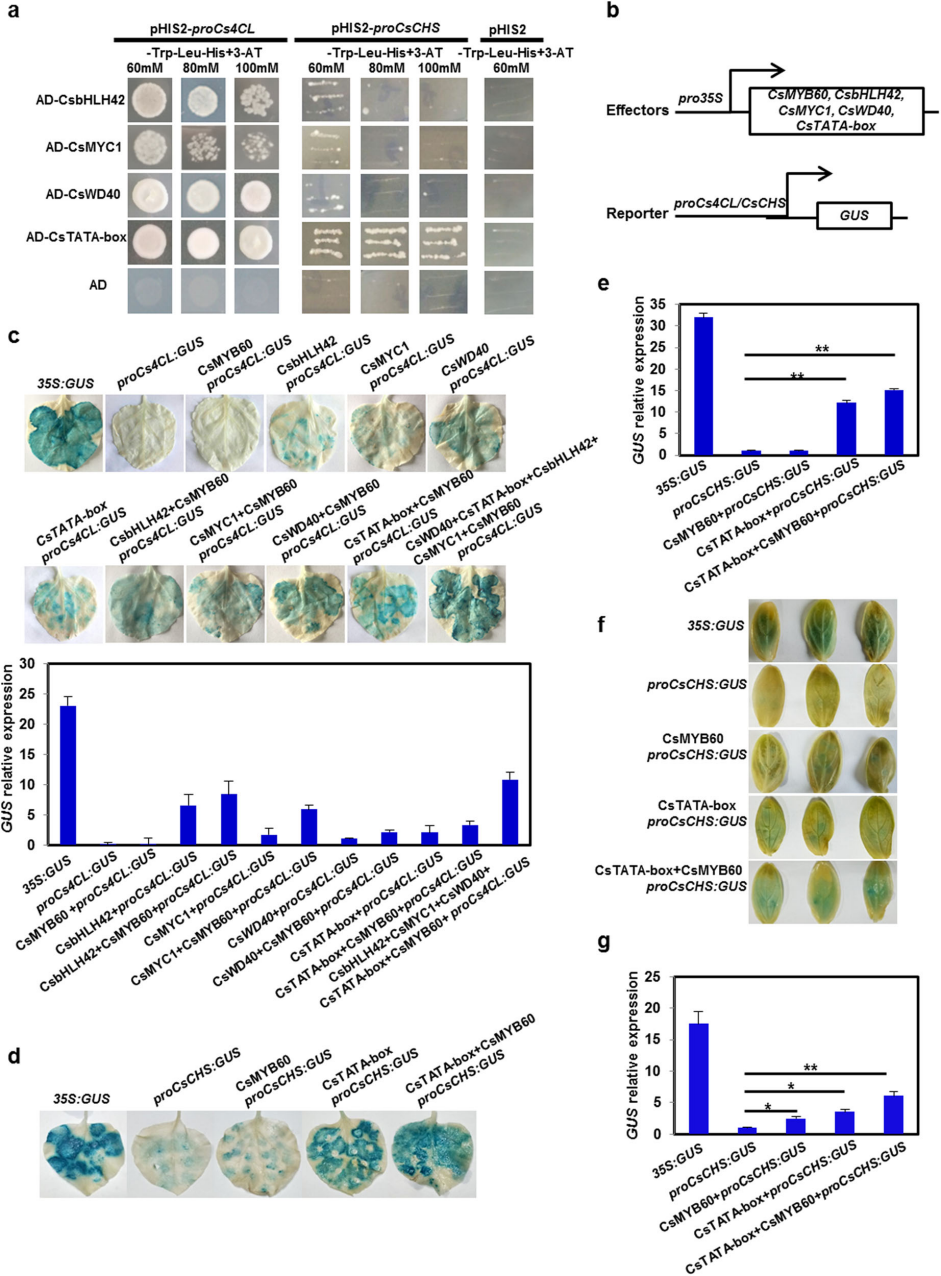
CsbHLH42, CsMYC1, CsWD40, and CsTATA-box binding protein all directly bound to the promoter of *Cs4CL*, but only CsTATA-box binding protein activated *CsCHS*. **a** Yeast-one-hybrid assays. **b** Schematic diagrams of the effector constructs and *GUS* reporter gene constructs driven by the *Cs4CL* promoter or *CsCHS* promoter. **c***GUS* activity assays in tobacco leaves showing that CsMYB60 promoted the expression of *Cs4CL*. **d***GUS* activity assays showing that CsTATA-box binding protein promoted the expression of *CsCHS* and that CsMYB60 increased CsTATA-box binding protein activation in tobacco leaves. **e** Relative *GUS* expression in the transient expression assays in (**d**). **f***GUS* activity assays showing that CsTATA-box binding protein promoted the expression of *CsCHS* and that CsMYB60 increased CsTATA-box binding protein activation in cucumber cotyledons. **g***GUS* relative expression in the transient expression assays in (**f**). Three biological replicates were performed for the gene expression analyses. Error bars represent SE. Statistical significance: *P < 0.05, **P < 0.01

CsMYB60 activated the expression of the key genes *Cs4CL* and *CsCHS* but did not directly bind to their promoters, indicating that the mode of activation was indirect (Figs. 3b and S4d). To test whether CsMYB60 regulates the expression of *Cs4CL* and *CsCHS* through its interacting proteins, we performed Y1H assays. We found that CsbHLH42, CsMYC1, CsWD40, and CsTATA-box binding protein could directly bind to the promoter of *Cs4CL*, but only CsTATA-box binding protein could bind to the promoter of *CsCHS* (Fig. 5a).

Transient expression assays in tobacco leaves or cucumber cotyledons showed that CsMYB60 could activate the expression of *Cs4CL* and *CsCHS* through its interacting proteins, which could directly bind to the *Cs4CL* and *CsCHS* promotors (Fig. 5). Although CsbHLH42, CsMYC1, CsWD40, and CsTATA-box binding protein could promote the expression of *GUS* driven by the *Cs4CL* promoter to a certain extent, when CsMYB60 was cotransfected with these proteins, *GUS* expression was increased in tobacco leaves. When the five proteins were coinjected, *GUS* expression reached a maximum^6^ (Fig. 5c). CsMYB60 could effectively increase the expression of *GUS* driven by the *CsCHS* promoter in cucumber cotyledons but had no obvious effect in tobacco leaves, suggesting that CsMYB60 also regulates *CsCHS* indirectly (Fig. 5d–g). Then, we found that CsTATA-box binding protein could activate the expression of *GUS* in cucumber cotyledons and tobacco leaves (Fig. 5d–g), and that the *GUS* expression level was significantly increased upon cotransfection with *35S*:*CsMYB60* and *35S*:*CsTATA-box* (Fig. 5d–g).

### CsMYC1 physically interacts with CsbHLH42, CsWD40, and itself

MBW complexes regulate flavonoid biosynthesis in numerous plant species, such as *Arabidopsis*, strawberry, and petunia^37,45,46^. To compare CsbHLH42, CsMYC1, and CsWD40 with the bHLH and WD40 TFs involved in flavonoid synthesis from other species, two phylogenetic trees were constructed (Fig. S10). The results showed that the CsbHLH42 and CsMYC1 proteins shared high similarities with bHLH TFs that regulate the biosynthesis of flavonoids in other species (Fig. S10a), whereas the CsWD40 protein shared low similarity with WD40 TFs from other species (Fig. S10b).

**Fig. 6:**
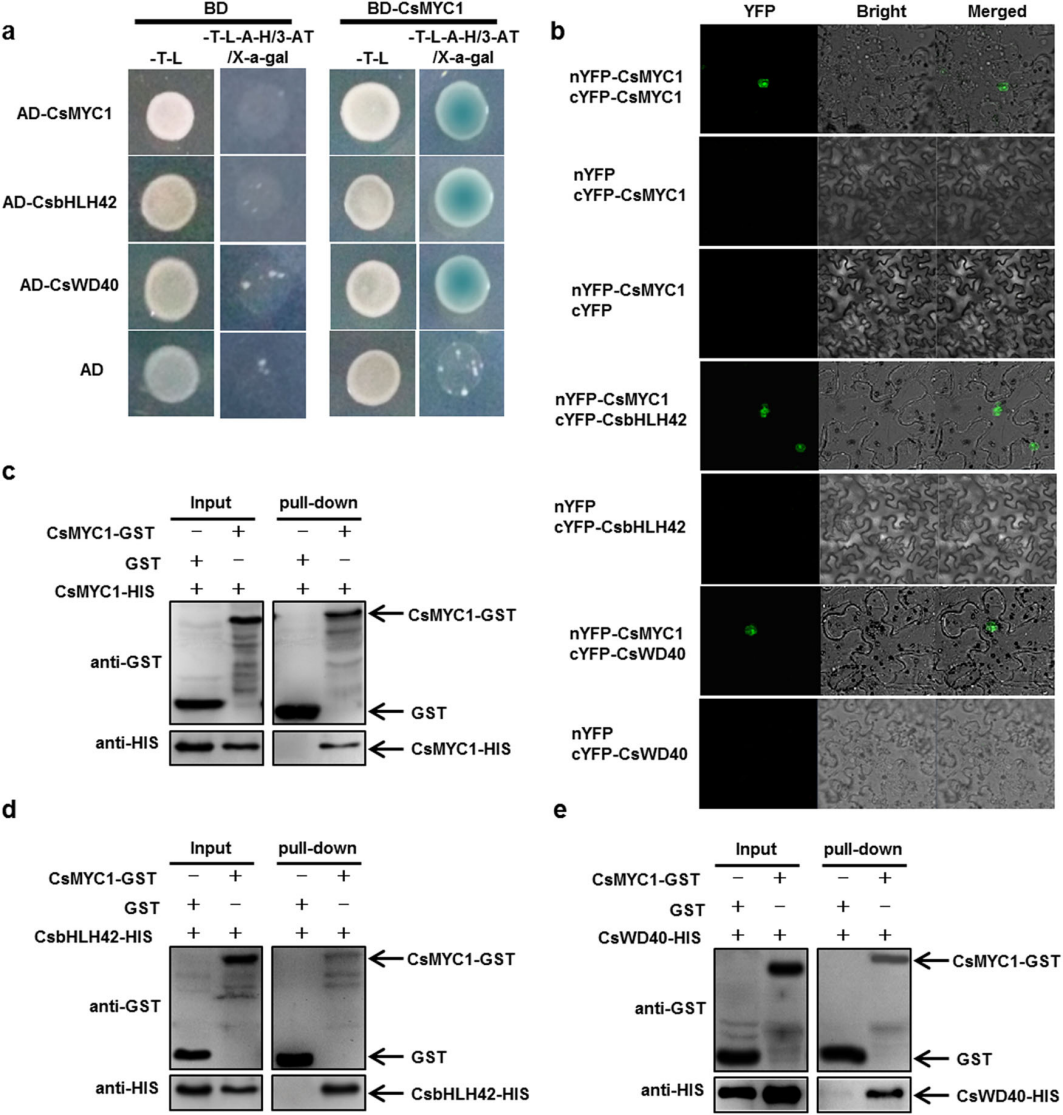
CsMYC1 interacted with CsbHLH42, CsWD40, and itself. **a** Yeast two-hybrid assays validating the interaction of CsMYC1 with itself, CsbHLH42, and CsWD40. **b** BiFC assays. **c**–**e** Pull-down assays

The Y2H results showed that CsMYC1 directly interacted with CsbHLH42, CsWD40, and itself but not with CsTATA-box binding protein (Fig. 6a). The results were further confirmed by BiFC (Fig. 6b) and pull-down assays (Fig. 6c–e).

### *CsbHLH42* and *CsMYC1* can induce the biosynthesis of anthocyanins and PAs in *Arabidopsis*

Although interacting relationships have been identified among these proteins, it is necessary to explore their biological functions. *Arabidopsis* was used for genetic transformation to clarify the biological functions of *CsbHLH42*, *CsMYC1, CsWD40*, and *CsTATA-box binding protein* (Figs. S11b and S12a). The visible pigmentation in the cotyledons and hypocotyls was significantly increased only in the *CsbHLH42*- and *CsMYC1*-overexpressing transgenic lines and not in the *CsWD40*- and *CsTATA-box*-overexpressing transgenic lines compared with the control (Fig. S11a). The intense pigmentation phenotype in the cotyledons and hypocotyls of the *CsbHLH42*- and *CsMYC1*-overexpressing lines is expected to result from the increases in anthocyanin and PA accumulation (Fig. S11c, d). However, neither anthocyanin nor PA contents were altered in the *CsWD40*- and *CsTATA-box*-overexpressing lines compared with the control (Fig. S12b, c).

Endogenous structural genes, including *AtPAL*, *AtCHS*, *AtDFR*, and *AtANS*, etc. were coordinately upregulated in the transgenic lines overexpressing *CsbHLH42* or *CsMYC1* (Fig. S11e). In addition, CsMYC1 and CsbHLH42 could interact with the MYB TFs AtPAP1 and AtTT2, which are involved in the regulation of flavonoid biosynthesis in *Arabidopsis* (Fig. S12d).

## Discussion

### *CsMYB60* is the *B* gene that controls the biosynthesis of flavonols and PAs in cucumber

As an economic trait, the spine color of cucumber fruit was investigated in a classical genetics study one century ago^47^. Thereafter, it was confirmed that black spines were dominant over white spines and that this trait was controlled by a single gene, the *B* gene^48^. Although the *B* locus was found early last century^47,48^, its mapping and cloning were delayed for quite some time due to a lack of genomic information. In 2013, the *B* locus was finely mapped to a 50 kb genomic DNA region containing an R2R3-MYB TF-encoding gene, *CsMYB60*^41,42^. The best candidate gene for the *B* locus seemed to be *CsMYB60*, which controls the coloration of the orange mature fruit skins and black spines of cultivated cucumber^41,42^. Recent studies supported this hypothesis^6,49^. Moreover, the present study further confirmed this hypothesis using transgenic lines overexpressing *CsMYB60* (Fig. 2). In addition, it has been clarified that the main pigments of black spines are flavonols and PAs^6^. In addition to black spines and orange mature fruit skins, flavonols and PAs accumulated in other tissues at different levels (Figs. 1 and S1). Moreover, the distribution patterns of flavonols and PAs were similar to those in the black-spined inbred line (Figs. 1, S1, S4, and S5). Therefore, *CsMYB60* is the *B* gene that regulates the biosynthesis of flavonols and PAs not only in black spines and orange mature fruit skins but also in other tissues in cucumber.

Flavonols and PAs that exist in the seeds, bark, leaves, and fruit of various plants; not only do these compounds play important roles in many biological processes such as plant growth and development and resistance to insects and diseases^17,50^, but they also greatly benefit human health^8,9^. Flavonols and PAs showed high accumulation in the black spines and orange mature fruit skins of the black-spined inbred lines but presented low accumulation in other tissues^6,41^ (Figs. 1 and S1). This spatiotemporal pattern of flavonols and PAs in cucumber does not benefit human health because when cucumbers are consumed fresh, the spines are washed off, and the fruit skins contain very low levels of flavonols and PAs at the commercial stage^6^ (Fig. 1). The present study showed that the contents of flavonols and PAs could be increased in the fruit pericarp and pulp (Fig. S4a–c). Therefore, this study provides a theoretical basis, target genes, and technical support for the design of molecular breeding strategies to improve the fruit quality of cucumber.

### The mechanism of flavonol and PA biosynthesis regulated by CsMYB60 in cucumber

The regulation of flavonoid biosynthesis has been studied in depth, especially in the model plant *A. thaliana*^30,51^. Key structural genes and numerous transcription factors are required for flavonol and PA biosynthesis. Since the first plant MYB regulating anthocyanin biosynthesis was identified in maize (*Zea mays*)^52^, a growing body of evidence has demonstrated that MYB TFs are major regulators of flavonoid biosynthesis in diverse species^53,54^. In *Arabidopsis*, three closely related R2R3-MYB proteins, MYB11, MYB12, and MYB111, transcriptionally regulate flavonol biosynthesis by directly activating the early biosynthetic genes (EBGs) *CHS*, *CHI*, *F3H*, and *FLS*, whereas the MBW complex activates the late biosynthetic genes (LBGs) *DFR*, *LODX*, and *ANR*, leading to the production of PAs and anthocyanins^30^. The seed-specific accumulation of PAs requires the activity of the MBW complex composed of TT2, TT8, and TTG1, and anthocyanin biosynthesis is regulated by the MBW complex consisting of one R2R3-MYB TF (PRODUCTION OF ANTHOCYANIN PIGMENTS (PAP1), PAP2, MYB113, or MYB114), one bHLH protein (TT8, GLABROUS3 (GL3), or ENHANCER OF GLABRA3 (EGL3)) and TTG1^30^.

**Fig. 7:**
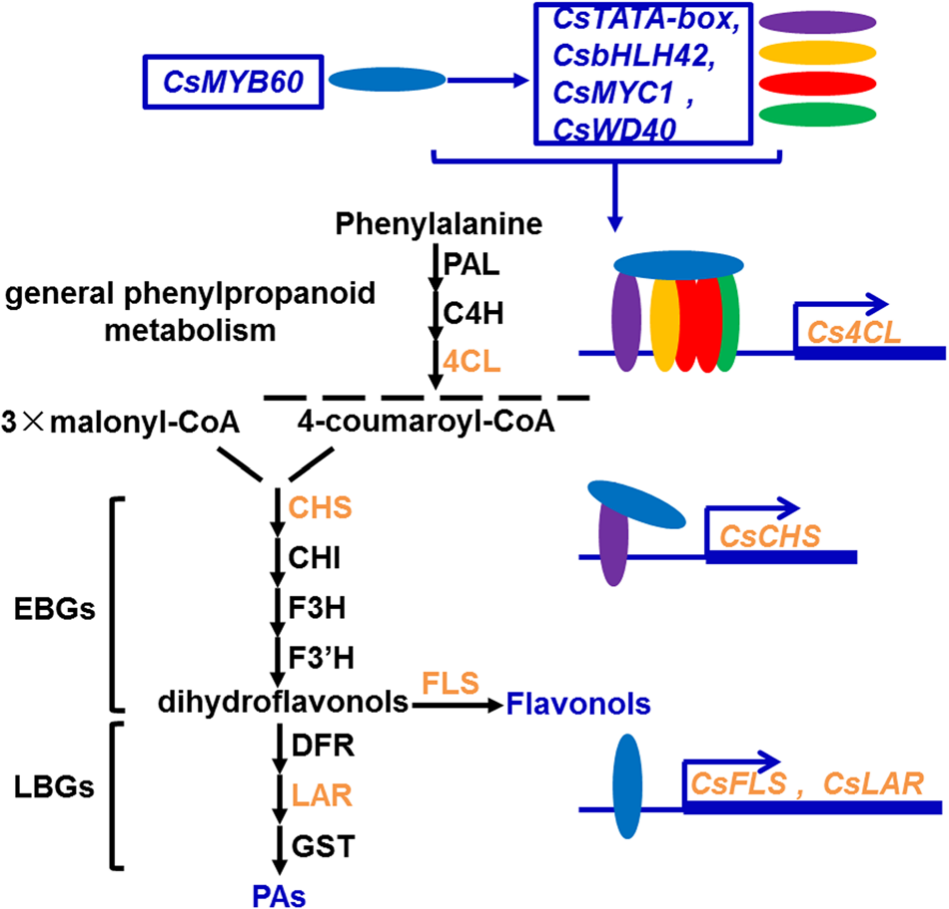
A schematic model for the mechanism of flavonoid biosynthesis regulated by CsMYB60 in cucumber. CsMYB60 could activate the expression of *CsFLS* and *CsLAR* by directly binding to their promoters. Additionally, CsMYB60 directly or indirectly activated the expression of Cs*bHLH42*, Cs*MYC1*, Cs*WD40*, and Cs*TATA-box binding protein*, whose products then interacted with CsMYB60 to form the complex regulating the expression of *Cs4CL*, and CsMYB60 interacted with CsTATA-box binding protein to promote the expression of *CsCHS*. Blue, orange, red, green, and purple ellipses represent CsMYB60, CsbHLH42, CsMYC1, CsWD40, and CsTATA-box binding protein, respectively

Although *CsMYB60* is known to be a key regulator of flavonoid biosynthesis^6^, it is still unclear how *CsMYB60* transcriptionally regulates the structural genes of the biosynthetic pathway of flavonoids in cucumber. In this study, we demonstrated the mechanism whereby flavonol and PA biosynthesis is regulated by *CsMYB60* (Fig. 7). The expression levels of some structural genes involved in flavonoid biosynthesis were increased in the *35S*:*CsMYB60* transgenic lines. For example, the expression levels of *CsCHS*, *CsFLS*, *CsLAR*, and *Cs4CL* in the transgenic lines were significantly higher than those in their nontransgenic siblings (Fig. S4d). CHS catalyzes the first committed step in flavonoid biosynthesis, and FLS and LAR are essential enzymes for the biosynthesis of flavonols and PAs, respectively, in plants^30–32^. However, these three key genes in flavonoid biosynthesis did not change white spines into black spines in a transient expression system, but *Cs4CL* did, which suggests that *Cs4CL* is the limiting gene for flavonoid biosynthesis in cucumber^6^. However, *CsCHS*, *CsFLS*, and *CsLAR* must work in coordination with *Cs4CL* (Fig. S4d); moreover, this coordination could be executed by *CsMYB60* (Fig. 7). As a homolog of AtMYB11, 12, and 111 (Fig. S2), which directly regulate EBGs^30^, *CsMYB60* could directly activate the expression of *CsFLS* and *CsLAR* (Figs. 3 and S6). *CsMYB60* did not directly promote the expression of *CsCHS*, a key gene in flavonoid biosynthesis (Fig. 5e), but did increase the direct activation activity of CsTATA-box binding protein toward *CsCHS* through the protein interaction between CsMYB60 and CsTATA-box binding protein (Figs. 4a, b, f and 5). In addition to the EBGs and LBGs involved in flavonoid biosynthesis, CsMYB60 could indirectly regulate *Cs4CL*^6^, the gene responsible for the last step of the general phenylpropanoid metabolism pathway, through its interacting proteins CsbHLH42, CsMYC1, CsWD40, and CsTATA-box binding protein, which can directly bind to the *Cs4CL* promotor to activate its expression (Figs. 4 and 5). Beyond the MBW complex, we identified a new function of the CsTATA-box binding protein in the regulation of flavonoid biosynthesis, which has never been reported previously to our knowledge. Therefore, there might be a unique regulatory mechanism for flavonoid biosynthesis in cucumber involving CsMYB60 as a key regulator. Although MYBs are common key factors in the regulation of flavonoid accumulation in plants, most species present distinct regulatory mechanisms^53,54^. However, further studies are needed to clarify what kind of complexes are formed during the activation of *Cs4CL* and what other factors take part in the fine-tuning of flavonoid biosynthesis in cucumber, which will reveal TFs that may be used as tools for metabolic engineering for flavonoid production in cucumber fruits.

## Materials and methods

### Plant materials and growth conditions

White-spined (RNS8) and black-spined (RNS9) cucumber inbred lines were used for flavonol and PA analyses^6^. The cucumber inbred line Xintaimici (wild type, WT) and the *Arabidopsis* Columbia-0 ecotype were used for genetic transformation. Two generations of cucumbers per year were grown in a solar greenhouse at the experimental station of Shandong Agricultural University, Tai’an, China.

### Construction of the *CsMYB60* overexpression vector and genetic transformation

The complete *CsMYB60* coding sequence was inserted into the BamHI and SacI sites of the pCAMBIA1300 vector to form the *35S:CsMYB60* construct. Subsequently, the *35S*:*CsMYB60* vector was transformed into Xintaimici^55^. Transgenic plants were screened on hygromycin-containing medium, and the presence of the transgene was confirmed by PCR with genomic DNA as the template.

### Spatial and temporal expression analysis of *CsMYB60* by qRT-PCR

Total RNA was isolated from cucumber and *Arabidopsis* plants using an RNAprep pure Plant Kit (TianGen, Beijing, China) following the manufacturer’s instructions. Subsequently, the RNA was reverse transcribed using the PrimeScript^®^ 1st Strand cDNA Synthesis Kit (Takara, Japan). qRT-PCR was performed using the UltraSYBR Mixture (with ROX I; Cwbiotech) in the iCycler iQ5 system (BioRad, CA, USA). The results were normalized to those of the cucumber *ACTIN* gene. Three biological replicates were performed for each analysis. The primers used in this study are provided in Table S1.

### Yeast two-hybrid (Y2H) screening and confirmation

Yeast (*Saccharomyces cerevisiae*) two-hybrid (Y2H) assays were performed as described in the manufacturer’s instructions (Clontech, Mountain View, CA, USA). The *CsMYB60* CDS was inserted into the bait vector pGBKT7. Subsequently, the CDSs of *CsbHLH42*, *CsMYC1*, *CsWD40*, and *CsTATA-box binding protein*, etc. were cloned into the prey vector pGADT7. All of the constructs were transformed into the yeast strain AH109 Gold using the lithium acetate method. Subsequently, the cells were cultured on synthetic defined (SD) medium lacking Trp and Leu (SD/-Trp-Leu) at 28 °C. Putative transformants were transferred to medium lacking Leu, Trp, His, and adenine (SD/-Leu-Trp-His-Ade) with 3-AT and X-a-gal.

### Electromobility shift assays

EMSAs were performed as previously described^56^. *CsMYB60* was cloned into the vector pGEX4T-1. The production of the recombinant CsMYB60-GST protein was induced in BL21 cells. Subsequently, the recombinant protein was purified using glutathione Sepharose beads (Thermo Scientific, Waltham, MA, USA). Three oligonucleotide probes for the *CsFLS* and *CsLAR* promoters were labeled using an EMSA probe biotin labeling kit (Beyotime, Shanghai, China). The EMSAs were carried out using an EMSA kit (Thermo Scientific) following the manufacturer’s instructions. Excess unlabeled oligonucleotides were also added to the reactions. The primers used in this study are provided in Table S1.

### Pull-down assays

The *CsbHLH42*, *CsMYC1*, *CsWD40*, and *CsTATA-box binding protein* open reading frames were introduced into the PET-32a vector containing a histidine (HIS) tag sequence. The ORF of *CsMYB60* was inserted into the PGEX-4T-1 plasmid containing a glutathione S-transferase (GST) tag sequence. Then, the recombinant plasmids were transformed into *Escherichia coli* BL21 (DE3) (TransGen Biotech, China) to induce the formation of fusion proteins. The pull-down assays were performed following the instructions of the Pierce GST Spin Purification Kit (Thermo Scientific). Then, bound proteins were detected by immunoblotting with anti-HIS and anti-GST antibodies.

### BiFC assays

The full-length *CsbHLH42*, *CsMYC1*, *CsWD40*, *CsTATA-box binding protein*, and *CsMYB60* genes were cloned into the plasmids pSPYCE-35S and pSPYNE-35S. *Agrobacterium tumefaciens* LBA4404 cells were transformed with the recombinant plasmids, and different combinations of *Agrobacterium* strains were cotransformed into tobacco leaves. After 48 h of coinfiltration, YFP fluorescence was detected using a confocal microscope with excitation at 488 nm.

### PA staining and determination

The DMACA reagent [1% (w/v) in ethanol:6 M HCl (1:1, v/v)] was used to stain different tissues of cucumber for 1 h. Tissue samples of equal weight were ground with liquid nitrogen and suspended in a 70% (v/v) acetone aqueous solution containing 0.1% (w/v) ascorbic acid; this procedure was repeated three times. The corresponding PA content of each tissue was extracted by the method described previously^57^.

### Yeast one-hybrid (Y1H) assays

The promoter fragments (2000 bp) of Cs*4CL*, Cs*FLS*, and Cs*LAR* were inserted into the pHIS2 plasmid. The *CsbHLH42*, *CsMYC1*, *CsWD40*, *CsTATA-box binding protein*, and *CsMYB60* CDSs were separately inserted into the pGADT7 vector (Clontech) to obtain the constructs AD-CsMYB60, AD-CsbHLH42, AD-CsMYC1, AD-CsWD40, and AD-CsTATA-box, respectively. Yeast strain Y187 cells harboring different combinations of recombinant plasmids were examined on SD/-Leu-Trp-His medium with an optimal concentration of 3-AT.

### Transient *GUS* activity assays

Cucumber cotyledons or tobacco leaves were used to conduct transient *GUS* activity assays. The promoters of *CsFLS*, *CsLAR*, *CsCHS*, and *Cs4CL* were inserted into pCAMBIA1300-*GUS* to activate the *GUS* reporter gene. The ORFs of *CsMYB60, CsbHLH42, CsMYC1*, *CsWD40*, and *CsTATA-box binding protein* were separately cloned into the pCAMBIA1300 plasmid to obtain the *35S*:*CsMYB60*, *35S*:*CsbHLH42*, *35S*:*CsMYC1*, *35S*:*CsWD40*, and *35S*:*CsTATA-box* recombinant plasmids. The different combinations were injected into cucumber cotyledons (8-day-old) or tobacco leaves (5-week-old) for *Agrobacterium*-mediated transformation. The injected tobacco or cucumber seedlings were grown for approximately 2 days under normal conditions. *GUS* activity was measured as previously described^6,58^.

### HPLC analysis of flavonols extracted from cucumber under optimum conditions

For the analysis of flavonols in various tissues of cucumber, 0.3 g tissue was ground in liquid nitrogen and extracted with MeOH:H_2_O (50:50 v/v) at 4 °C for 2 h with constant shaking, followed by sonication for 30 min at room temperature. The mixture was subsequently centrifuged at 12,000 rpm for 10 min. After centrifugation, the supernatant was collected, filtered through a 0.45 µm filter and stored at 4 °C until chromatographic analysis. The flavonol extracts obtained from cucumber were analyzed using an Agilent 1200 liquid chromatographic (LC) system equipped with an autosampler, quaternary pump, and diode-array detector (DAD) (Agilent Technologies, Waldrom, Germany). The sample (20 µL) was injected into the HPLC-ESI-QToF and separated in a Superspher RP C18 column (4 × 250 mm^2^, 4 µm; Agilent Technologies). Elution was performed via gradient elution with solvent systems A (TFA:FA:H_2_O/0.1:2:97.9) and B (TFA:FA:CAN:H_2_O/0.1:2:35:62.9) using the following conditions: 0–12 min, 30–48% B; 12–22 min, 48–55% B; 22–35 min, 55–60% B; 35–45 min, 60–30% B, with a flow rate of 0.8 mL/min. The ultraviolet–visible light detector wavelength was monitored at 365 nm.

### Determination of the total anthocyanin content

The total anthocyanin contents of the samples were extracted with the HCl–methanol method. A 0.5 g sample was ground in liquid nitrogen and then incubated with 5 mL of cold 1% (v/v) methanol–HCl in the dark for 24 h. The absorbance of the solution was measured at 650, 620, and 530 nm with a UV-1600 spectrophotometer. The anthocyanin content was calculated using the following formula: OD = (*A*_530_ − *A*_620_) − 0.1(OD_650_ − OD_620_).

## Supplementary information

Supplementary Information

## Data Availability

The data that support the results are included within the article and its Supplementary Information. Other relevant materials are available from the corresponding author upon reasonable request.
