# Supplementary material for: CsMYB60 directly and indirectly activates structural genes to promote the biosynthesis of flavonols and proanthocyanidins in cucumber
Source: Hortic Res. 2020 Jul 1;7:103. doi: 10.1038/s41438-020-0327-z (PMC7327083; doi:10.1038/s41438-020-0327-z)
Supplement: Supplementary file 1 — Supplementary Information [file 41438_2020_327_MOESM1_ESM.docx]

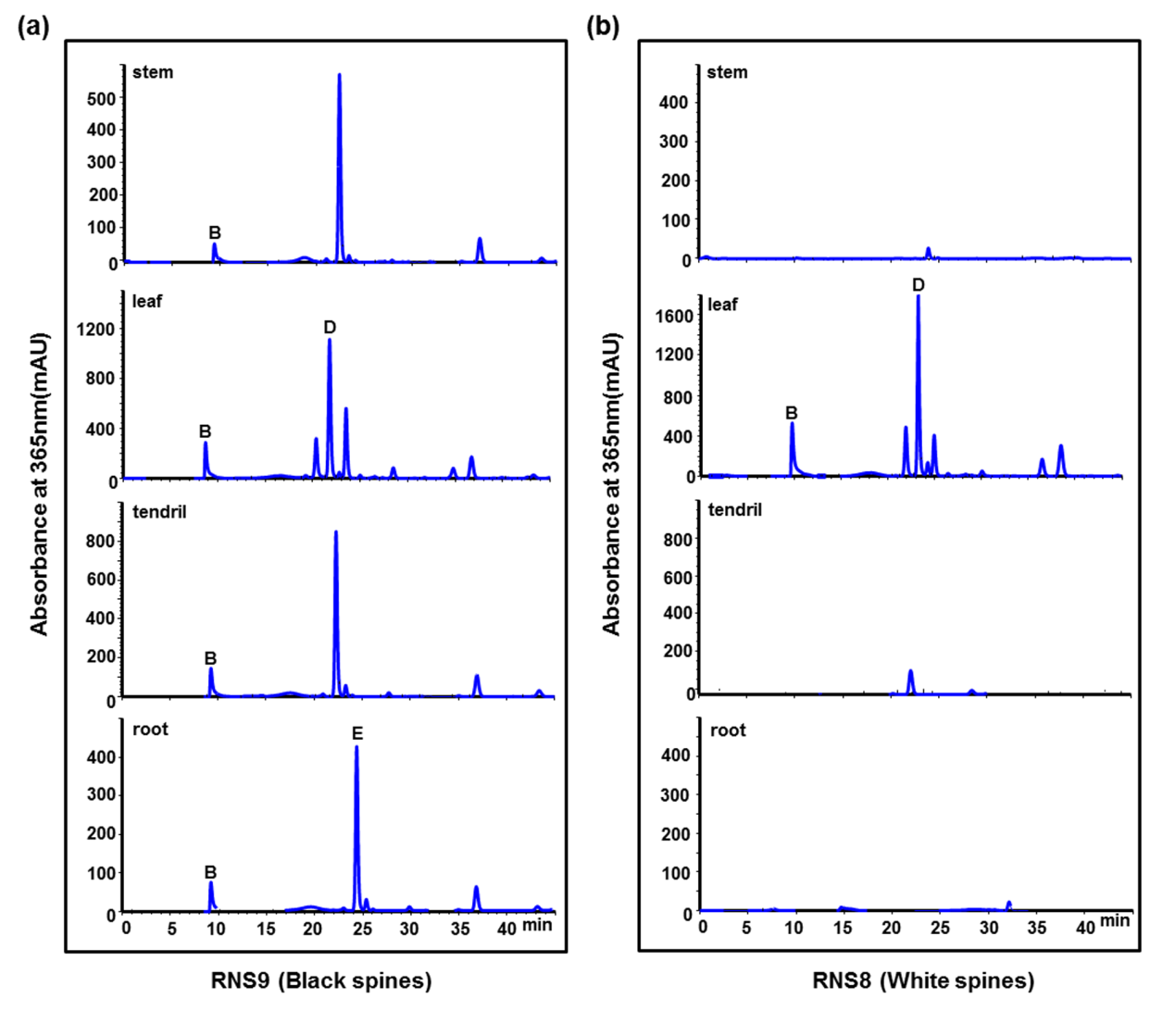


**Fig. S1 HPLC analyses of ﬂavonols in different tissues of RNS9 (black spines) and RNS8 (white spines) cucumber inbred lines.**

(a,b) HPLC analysis for the samples from black-spined and white-spined cucumber, respectively. All peaks were detected at UV absorbance of 365 nm. B: kempferol-3-O-rutinoside-7-O-glucoside, D: kaempferol-3-Orutinnoside and E: isorhamnetin-3-O-rutinoside.


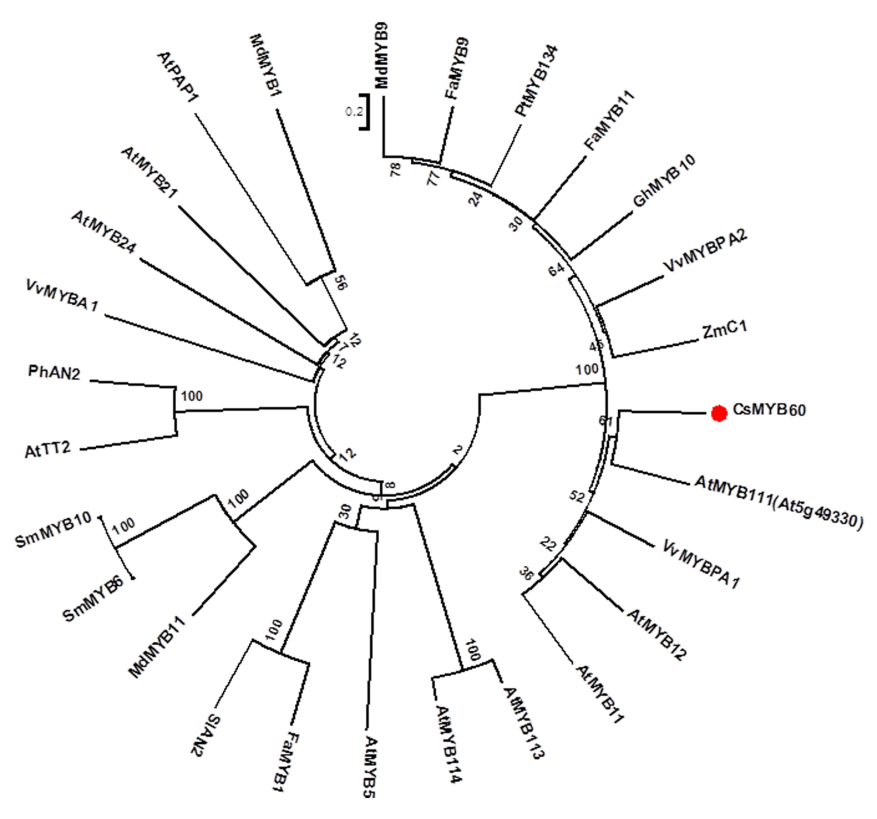


**Fig. S2 Phylogenetic analysis of MYB proteins.**

Phylogenetic relationship of MYB transcription factors related to flavonoid biosynthesis in different species. Protein names were obtained from GenBank with the accession numbers: CsMYB60 (CsaV3_4G001130.1); AtMYB5 (NP_187963.1); FaMYB11 (JQ989282.1); AtTT2 (NP_198405.1); MdMYB9 (DQ267900.1); FaMYB9 (JQ989281.1); AtMYB113 (AY519566.1); AtPAP1 (AY519563); AtMYB111 (AF371977); AtMYB11 (NM_116126); AtMYB12 (AF062864); AtMYB114 (AY008379.1); PhAN2 (AF146702.1); ZmC1 (NM_001112540.1); PtMYB134 (FJ573151.1); VvMYBPA1 (NP_001268160.1); VvMYBPA2 (NP_001267953.1); MdMYB11 (DQ074463.1); GhMYB10 (LOC107951064); FaMYB1 (AAK84064.1); SlAN2 (NP_001265992.1); SmMYB6 (AIP93873.1); SmMYB10 (AMK01804.1); MdMYB1 (NC_024239.1); AtMYB21 (NP_189418.2); AtMYB24 (NP_198851.1); VvMYBA1 (XP_010664911.1). The phylogenetic tree was constructed by the neighbor-joining method with 1,000 bootstrap replicates by the software MEGA6.0 (http://www.megasoftware.net/). The red circle represented CsMYB60.


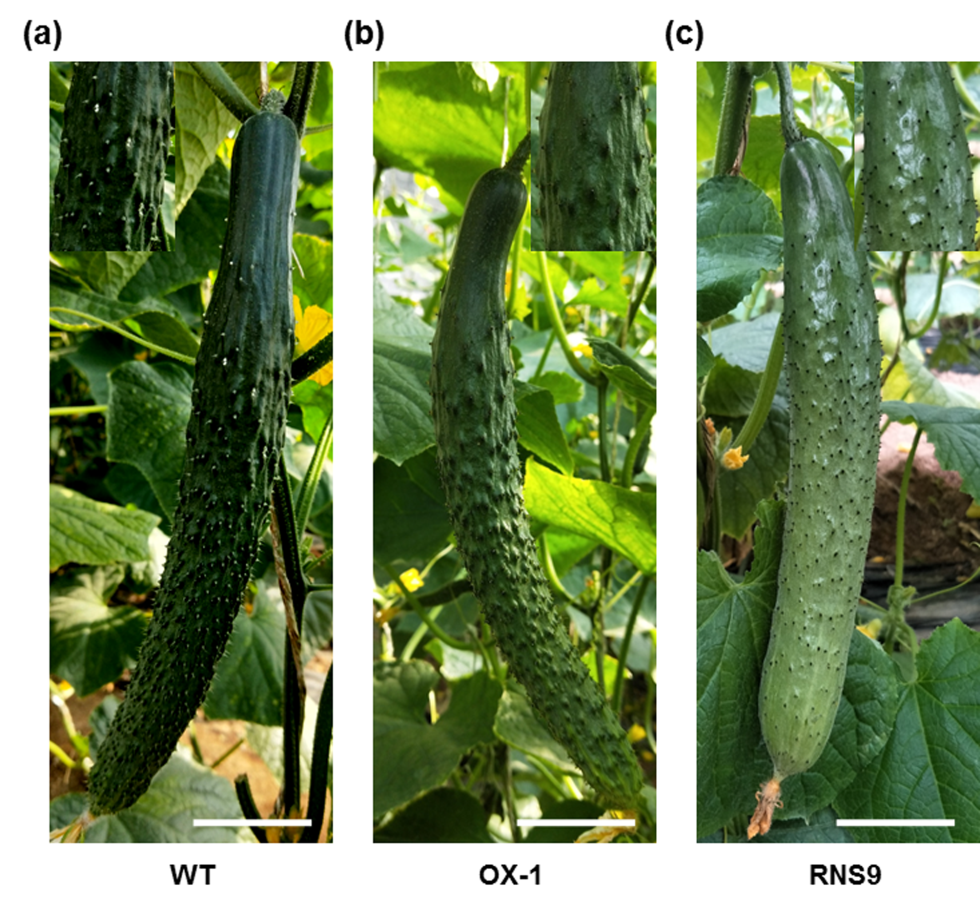


**Fig. S3 The commercial fruits at 12 DAA from WT (a), OX-1 (b) and RNS9 (c).** DAA, days after anthesis. Scale bars: (a-c) = 0.5 cm.


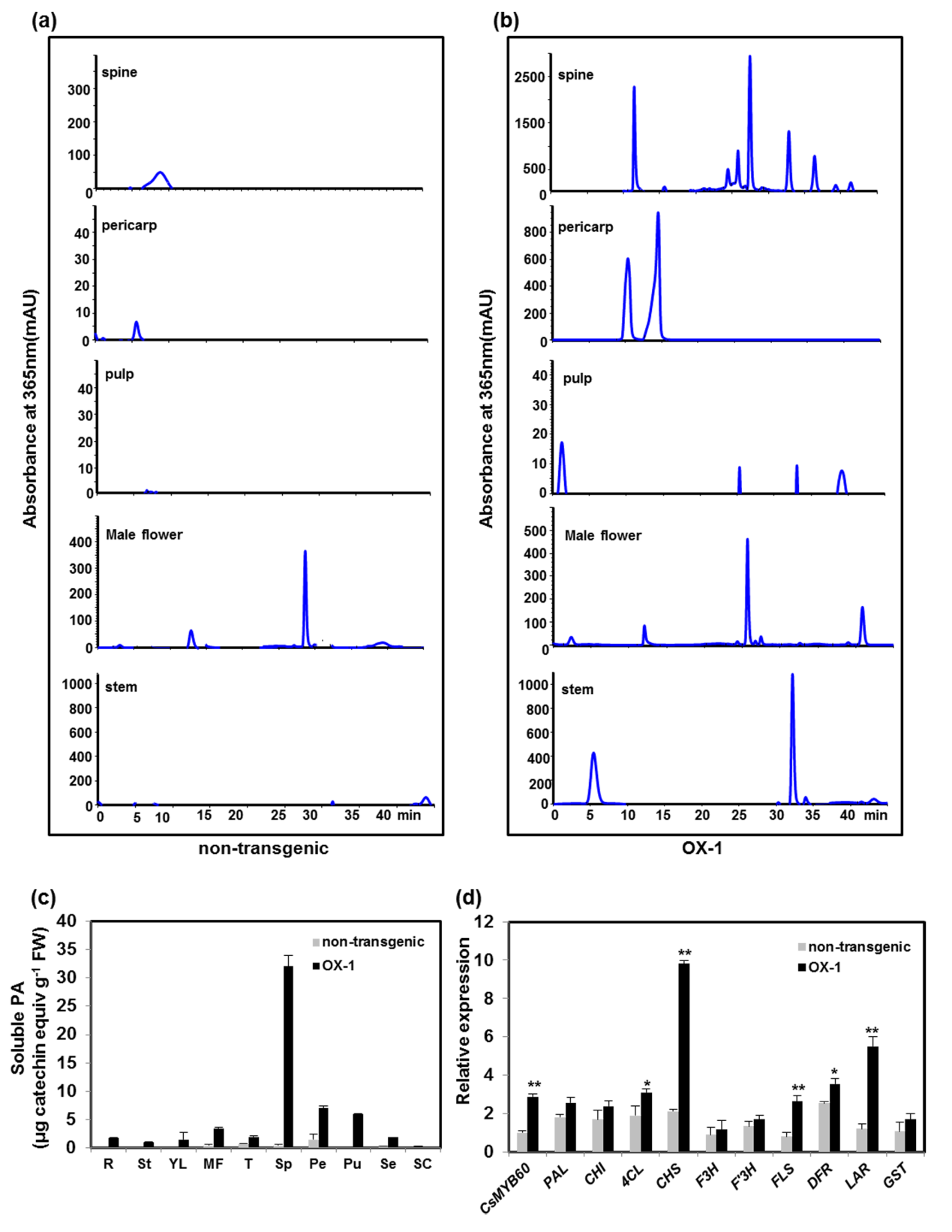


**Fig. S4 Levels of flavonols and PAs and the relative expression of flavonoid biosynthesis related genes in OX-1 and its non-transgenic siblings.** (a-b) HPLC analysis of flavonols in different tissues from non-transgenic siblings and OX-1. Absorbance was monitored at 365 nm. (c) Levels of soluble PAs in different tissues from OX-1 and its non-transgenic siblings using DMACA staining and quantification based on spectrophotometric absorbance. R, root; St, stem; YL, young leaf; MF, male flower; Sp, spine; T, tendril; Pe, pericarp; Pu, pulp; Se, seed; SC, seed coat. (d) The relative expression of *CsMYB60* and some structural genes in the flavonoid biosynthesis in OX-1 and its non-transgenic siblings. The cucumber *β-actin* gene was used as an internal control for normalization, and three biological replicates were used for these experiments. Error bars indicated SE. Significant differences were determined by Student’s *t*-test (*P < 0.05, **P < 0.01).


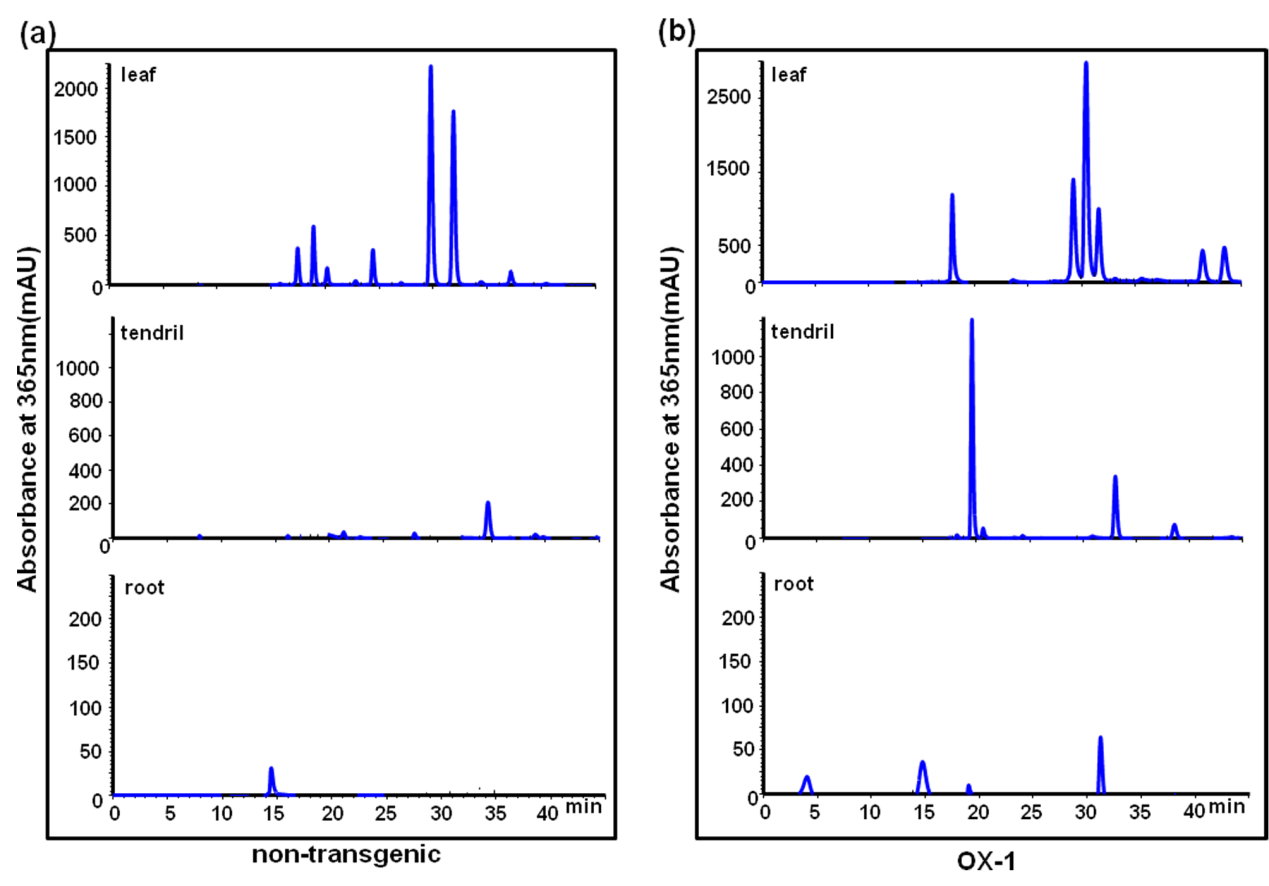


**Fig. S5 HPLC analyses of ﬂavonols in different tissues of the non-transgenic siblings (a) and OX-1 (b).** The non-transgenic siblings were isolated from T1 selfing progenies of OX1.


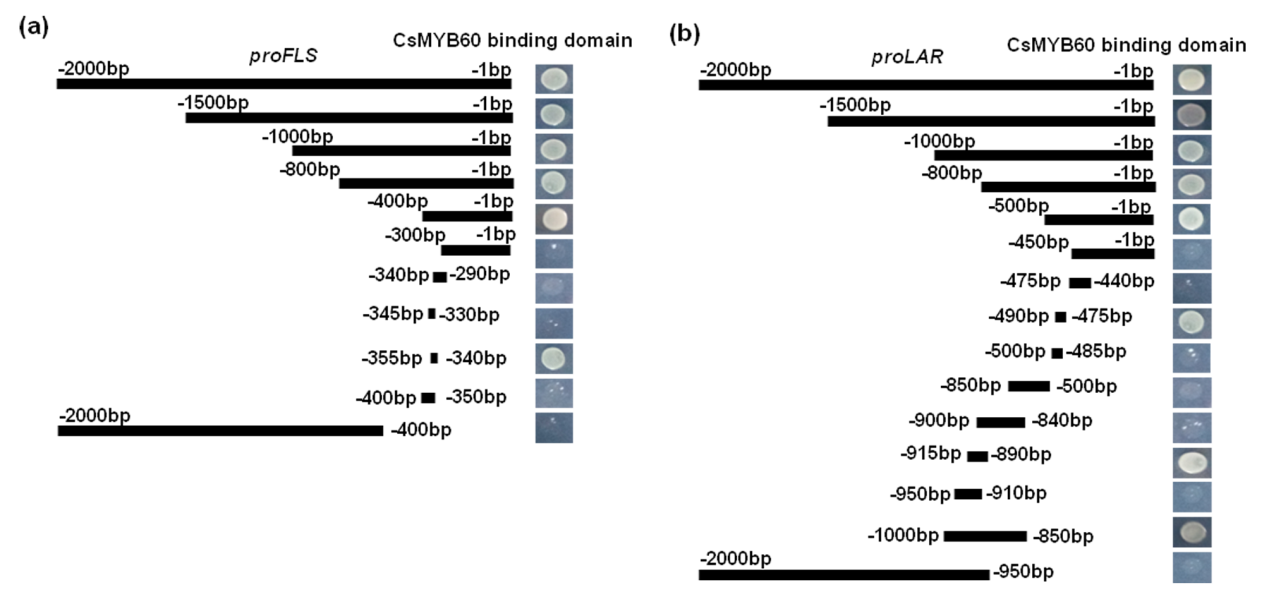


**Fig. S6 The yeast one-hybrid assay clarifying the *cis*-elements that CsMYB60 bound to the promoters of *CsFLS* (a) and *CsLAR* (b).**

**
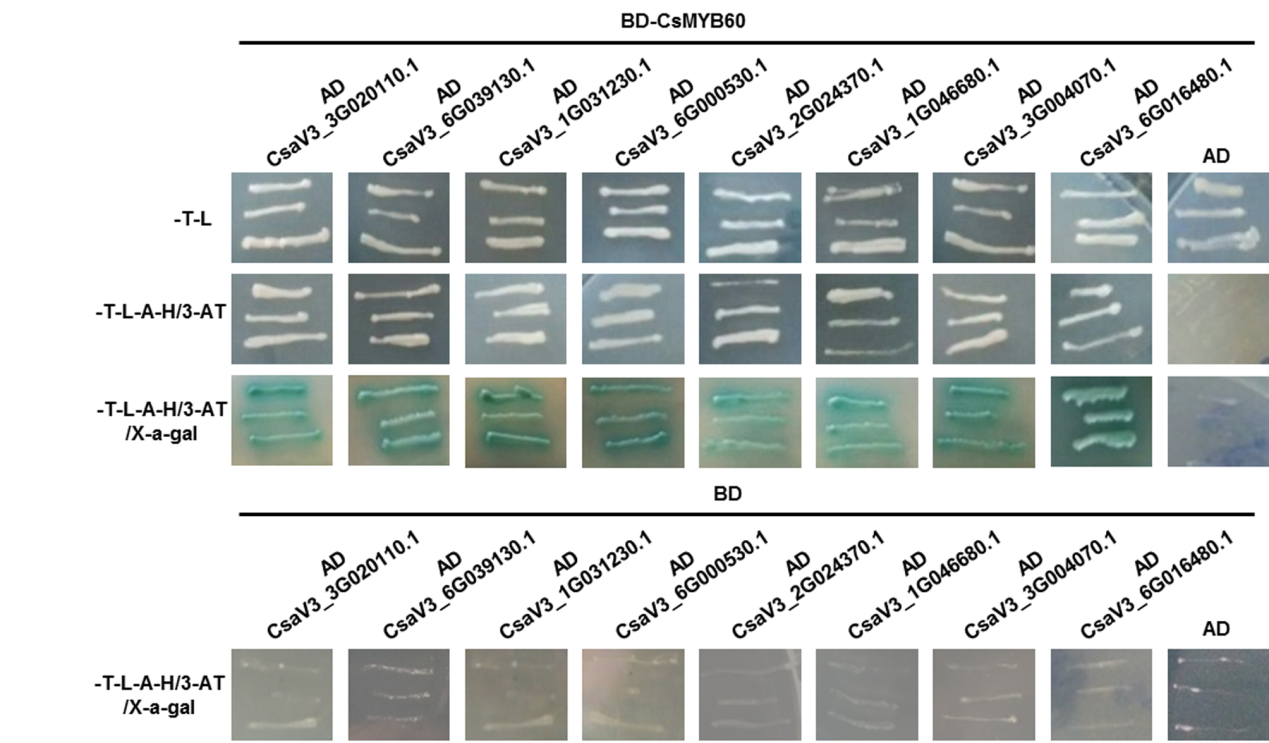
**

**Fig. S7 Yeast-two-hybrid assays validating the interaction of CsMYB60 with the screened putative interacting proteins.**


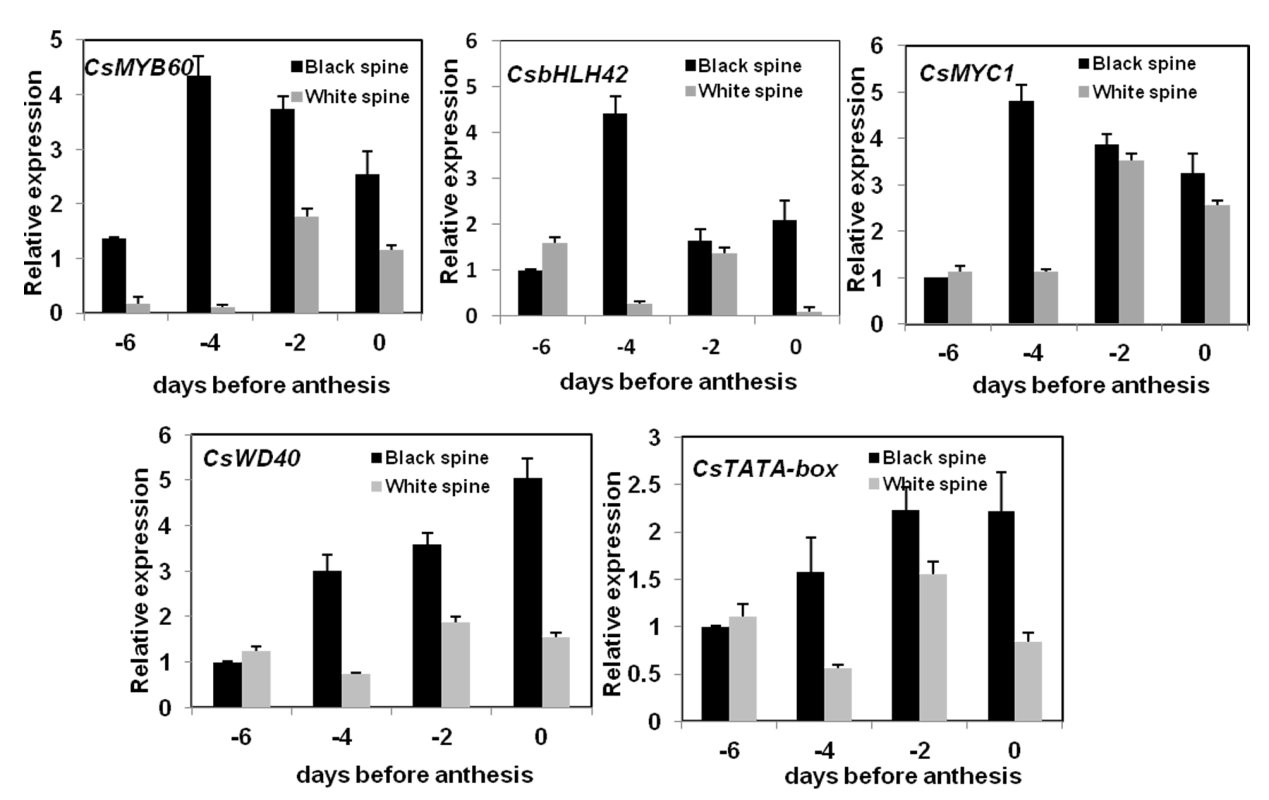


**Fig. S8 The qRT-PCR analysis of *CsMYB60, CsbHLH42, CsMYC1, CsWD40* and *CsTATA-box* *binding protein* in the spines from the black-spined and white-spined lines.**


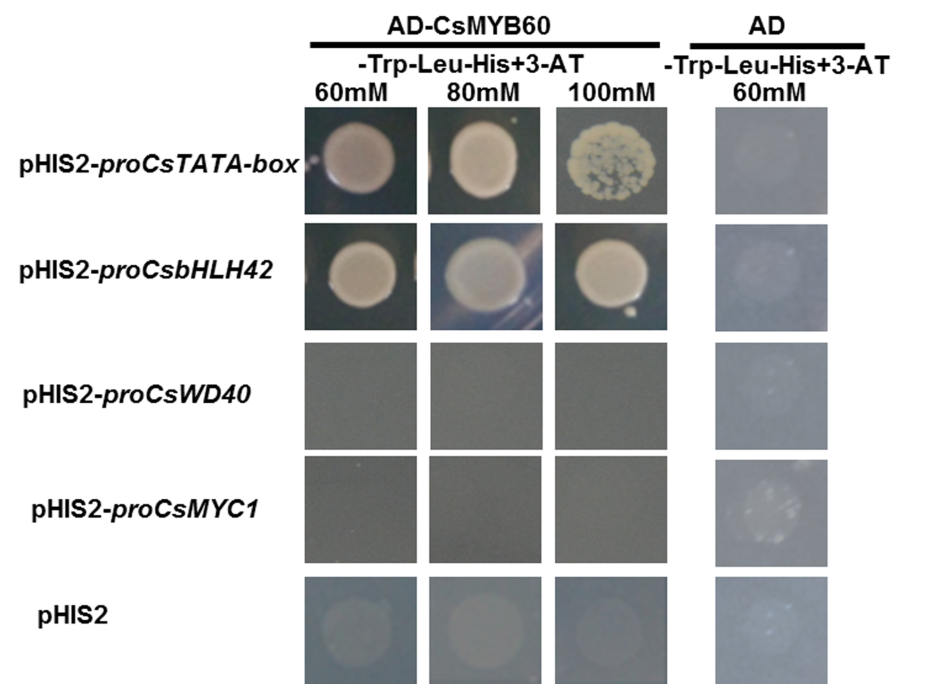


**Fig. S9 Yeast-one-hybrid assays showing that CsMYB60 could bind to the promoters of *CsbHLH42* and *CsTATA-box*** ***binding protein*, but not that of *CsMYC1* and *CsWD40*.** 60, 80 and 100mM, 3-AT concentration.


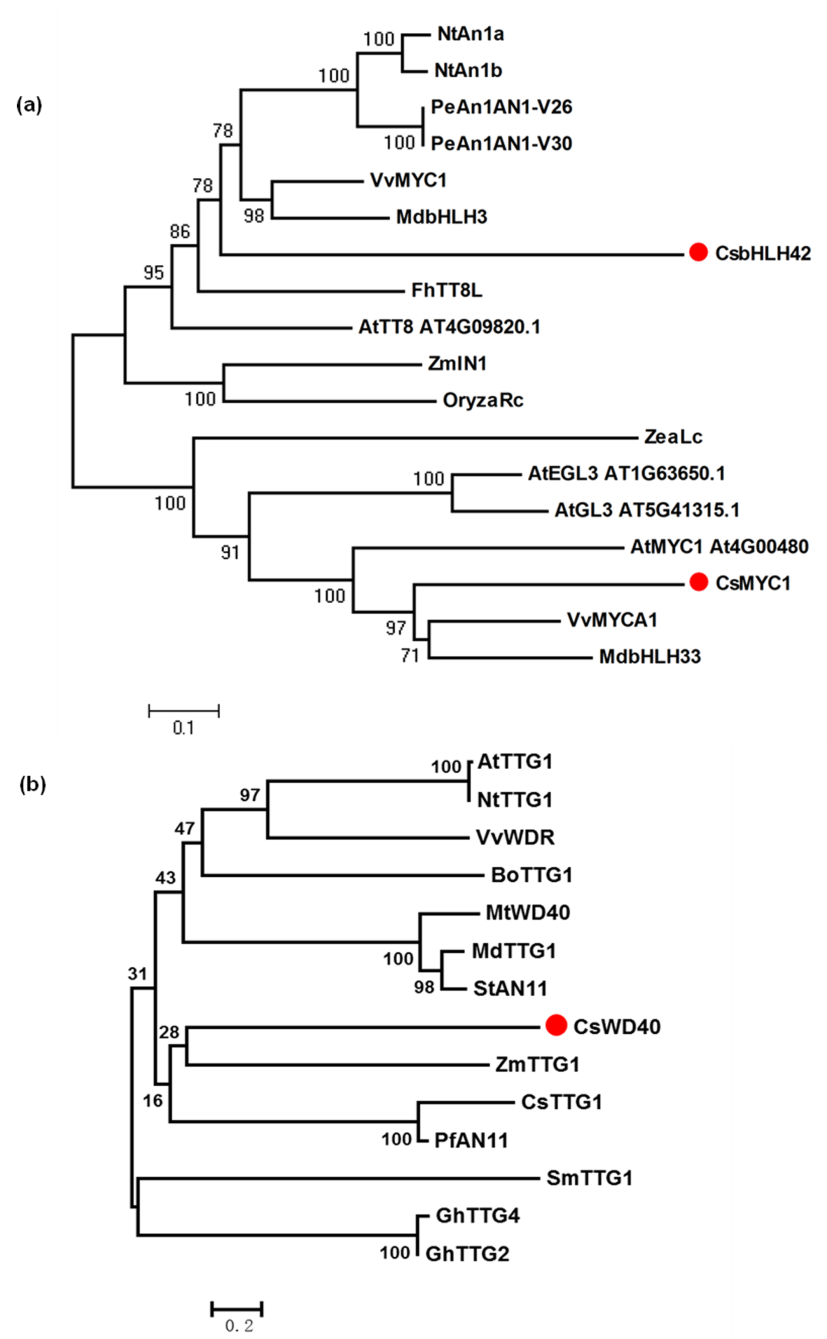


**Fig. S10 Phylogenetic relationship of bHLH (a) and WD-40 (b) transcription factors related to flavonoid biosynthesis in different species.**

(a,b) Phylogenetic tree was constructed using the neighbor-joining method by the MEGA6 software. The reliability of the trees was tested using a bootstrapping method with 1000 replicates. Numbers indicated bootstrap values for 1000 replicates. CsbHLH42 (CsaV3_6G037080.1), CsMYC1 (CsaV3_6G000530.1) and CsWD40 (CsaV3_1G031140.1) were indicated with red circles, respectively. The GenBank accession numbers of the bHLH protein sequences were as follows: Vitis vinifera VvMYC1 (EU447172), VvMYCA1 (EF193002); Oryza sativa OsRc (BAF42667); Zea mays ZmLc (AAA33504), ZmIN1 (AAB03841); Malus domestica MdbHLH33 (ABB84474), MdbHLH3(ADL36597.1); Arabidopsis thaliana AtTT8 (Q9FT81), AtEGL3 (Q9CAD0), AtGL3 (NP_680372), AtMYC1 (NC_003075.7); Petunia x hybrida PhAn1AN1-26 and PhAn1AN1-30 (AAG25927.1); Nicotiana tabacum NtAn1a (AEE99257.1), NtAn1b (AEE99258.1); Freesia hybrid cultivar FhTT8L (ATL14539.1). The GenBank accession numbers of the WD-40 protein sequences were as follows: Arabidopsis thaliana AtTTG1 (NP851069); Zea mays ZmTTG1(AIB05885.1); Solanum tuberosum StAN11 (AEF01097.1); Malus domestica MdTTG1 (AAF27919); Vitis vinifera VvWDR (ABF66625); Brassica oleracea BoTTG1 (ADP38078.1); Medicago truncatula MtWD40 (ABW08112); Perilla frutescens PfAN11 (BAB58883); Cucumis sativus CsTTG1 (ABY64743); Nicotiana tabacum NtTTG1 (ACJ06978.1); Saussurea medusa SmTTG1(ACN58396); Gossypium hirsutum GhTT2 (NP_001313775.1), GhTT4 (NP_016693970.1).


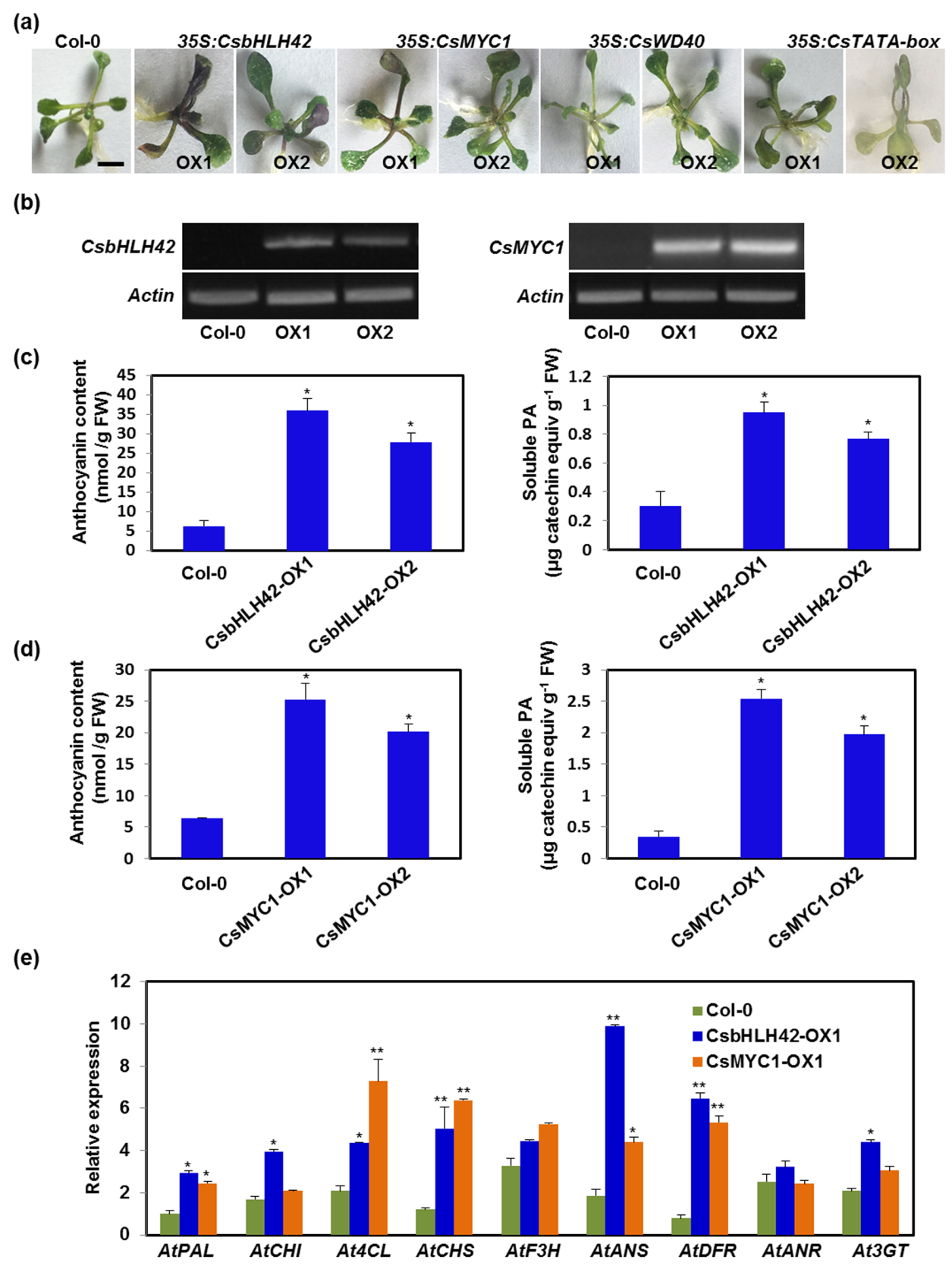


**Fig. S11 *CsbHLH42* and *CsMYC1* could regulate anthocyanin and PA accumulation in *Arabidopsis*.** (a) Phenotypes of the control Col-0 and *CsbHLH42*, *CsMYC1*, *CsWD40* and *CsTATA-box* *binding protein* transgenic *Arabidopsis* lines. Scale bar in (a) is 2 mm. (b) RT-PCR analysis of the *CsbHLH42* and *CsMYC1* genes in the control and transgenic lines. (c-d) Anthocyanin and PA contents in *Arabidopsis* seedlings from *CsbHLH42* (c) and *CsMYC1* (d) transgenic lines with Col-0 as the control. FW, fresh weight. (e) The qRT-PCR analysis of the expression of *Arabidopsis* endogenous structural genes involved in flavonoid biosynthesis in the Col-0, *CsbHLH42* and *CsMYC1* transgenic lines. The *ACTIN8* gene was used as an internal control for normalization, and three biological replicates were used for these experiments. Error bars indicated standard errors (SE). Significant differences were determined by Student’s t-test (*P < 0.05, **P < 0.01).


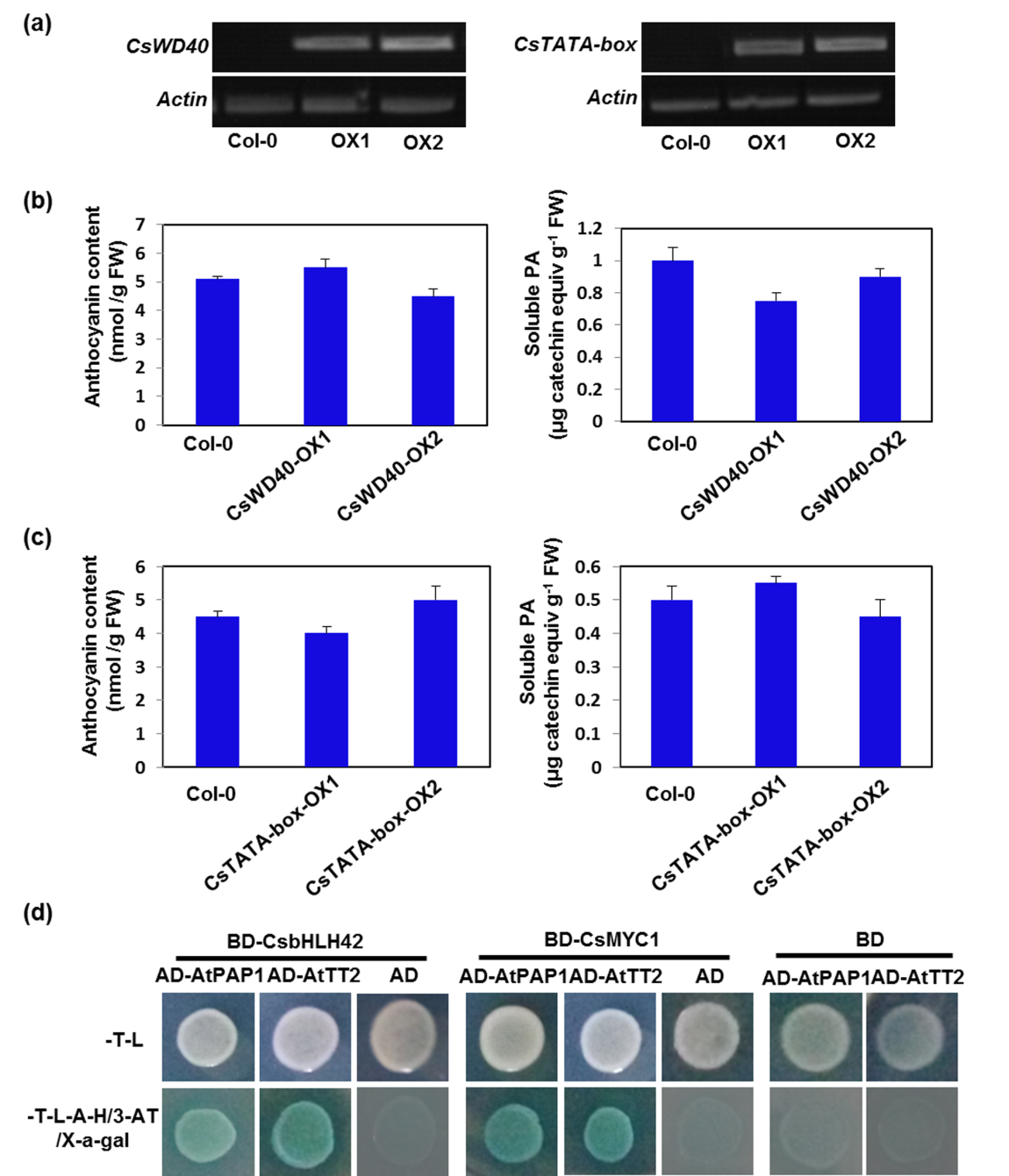


**Fig. S12 CsWD40 and CsTATA-box binding protein could not promote the flavonoid biosynthesis, and CsbHLH42 and CsMYC1 might regulate the flavonoid biosynthesis through interacting with AtPAP1 or AtTT2 in *Arabidopsis*.**

(a) The RT-PCR analysis of the *CsWD40* and *CsTATA-box* transgenic *Arabidopsis* lines. (b,c) Anthocyanin and PA contents of the *Arabidopsis* seedlings overexpressing *CsWD40* and *CsTATA-box* *binding protein* with Col-0 as the control. FW, fresh weight. (d) Y2H showing that CsbHLH42 and CsMYC1 could interact with AtPAP1 or AtTT2, respectively.

| **Table S1 Primers used in this study.** | |
| --- | --- |
| **Primer** | **Sequence (5'-3')** |
| RT-CsMYB60-F | GACGAGATGTCGTTGGACTTGG |
| RT-CsMYB60-R | CTATGTCCCCCGCGAAGCTTTC |
| RT-CsbHLH42-F | TGTTGGAGCTTCAGTGCCCATAT |
| RT-CsbHLH42-R | TCAAGTATCAACACAGGGTATG |
| RT-CsMYC1-F | CGACTCCTGCACGAAAAAT |
| RT-CsMYC1-R | ATCTGAAGTCTGCTCCACCATG |
| RT-CsWD40-F | GAGAGGATATGTGGATCCATG |
| RT-CsWD40-R | TGAGACCTCATCGGCACTTAC |
| RT-CsTATA-box-F | GATATTTGCATCGGGAAAGATG |
| RT-CsTATA-box-R | CCAGGAAATAACTCTGGTTCG |
| AD/BD-CsMYB60-F | GGCCATTACGGCCATGGGGAGAAGGCCGTGCTGTG(Sfil) |
| AD/BD-CsMYB60-R | GGCCGAGGCGGCCTCATGACAAAAGCCAAGAAATC(Sfil) |
| AD/BD-CsbHLH42-F | GGCCATTACGGCCATGGATTCCGACAGCTCGACC(Sfil) |
| AD/BD-CsbHLH42-R | GGCCGAGGCGGCCTCAAGTATCAACACAGGGTATG(Sfil) |
| AD/BD-CsMYC1-F | GGCCATTACGGCCATGGCTTGCGAACCTGGGTTTC(Sfil) |
| AD/BD-CsMYC1-R | GGCCGAGGCGGCCTCAGCTCTTGTTGACAACTTTC(Sfil) |
| AD/BD-CsWD40-F | GGCCATTACGGCCATGGGTTTTATGTTGACGC(Sfil) |
| AD/BD-CsWD40-R | GGCCGAGGCGGCCTCACTCATTTCCTGTCTCCTCC(Sfil) |
| AD/BD-CsTATA-box-F | GGCCATTACGGCCATGGCAGATCAAGGCTTGG(Sfil) |
| AD/BD-CsTATA-box-R | GGCCGAGGCGGCCTCATTGCTGGTTTTTCCTG(Sfil) |
| n/cYFP-CsMYB60-F | GGATCCATGGGGAGAAGGCCGTGCTGTG(BamHI) |
| n/cYFP-CsMYB60-R | CTCGAGTCATGACAAAAGCCAAGAAATC(Xhol) |
| n/cYFP-CsbHLH42-F | ACTAGTATGGATTCCGACAGCTCGACC(spel) |
| n/cYFP-CsbHLH42-R | CTCGAGTCAAGTATCAACACAGGGTATG(Xhol) |
| n/cYFP-CsMYC1-F | ACTAGTATGGCTTGCGAACCTGGGTTTC(spel) |
| n/cYFP-CsMYC1-R | CTCGAGTCAGCTCTTGTTGACAACTTTC(Xhol) |
| n/cYFP-CsWD40-F | ACTAGTATGGGTTTTATGTTGACGCTTT(spel) |
| n/cYFP-CsWD40-R | CTCGAGTCACTCATTTCCTGTCTCCTCC(Xhol) |
| n/cYFP-CsTATA-box-F | ACTAGTATGGCAGATCAAGGCTTGGAAGG(spel) |
| n/cYFP-CsTATA-box-R | CTCGAGTCATTGCTGGTTTTTCCTGAAC(Xhol) |
| CsMYB60-GST-F | CCCGGGTATGGGGAGAAGGCCGTGCTGTG(Smal) |
| CsMYB60-GST-R | CTCGAGTGACAAAAGCCAAGAAATCACCG(Xhol) |
| CsMYB60-His-F | GAGCTCATGGGGAGAAGGCCGTGCTG(Sacl) |
| CsMYB60-His-R | CTCGAGATGACAAAAGCCAAGAAATCACCG(Xhol) |
| CsbHLH42-GST-F | CCCGGGTATGGATTCCGACAGCTCGACC(Smal) |
| CsbHLH42-GST-R | CTCGAGAGTATCAACACAGGGTATGAT(Xhol) |
| CsbHLH42-His-F | GAGCTCATGGATTCCGACAGCTCG(Sacl) |
| CsbHLH42-His-R | CTCGAGAAGTATCAACACAGGGTATGATTTG(Xhol) |
| CsMYC1-GST-F | CCCGGGTATGGCTTGCGAACCTGGGTTTC(Smal) |
| CsMYC1-GST-R | CTCGAGGCTCTTGTTGACAACTTTCAAAAG(Xhol) |
| CsMYC1-His-F | GAGCTCATGGCTTGCGAACCTGGGTTTC(Sacl) |
| CsMYC1-His-R | CTCGAGAGCTCTTGTTGACAACTTTCAAAAG(Xhol) |
| CsWD40-GST-F | CCCGGGTATGGGTTTTATGTTGACGCTTTT(Smal) |
| CsWD40-GST-R | CTCGAGCTCATTTCCTGTCTCCTCCTTCTC(Xhol) |
| CsWD40-His-F | GAGCTCATGGGTTTTATGTTGACGCTTT(Sacl) |
| CsWD40-His-R | CTCGAGACTCATTTCCTGTCTCCTCCTTCTC(Xhol) |
| CsTATA-box-GST-F | CCCGGGTATGGCAGATCAAGGCTTGGAAGG(Smal) |
| CsTATA-box-GST-R | CTCGAGTTGCTGGTTTTTCCTGAACTCTGT(Xhol) |
| CsTATA-box-His-F | GAGCTCATGGCAGATCAAGGCTTGGAAG(Sacl) |
| CsTATA-box-His-R | CTCGAGATTGCTGGTTTTTCCTGAACTCTGT(Xhol) |
| *35S*:CsMYB60-F | GGATCCATGGGGAGAAGGCCGTGCTGTG(BamHI) |
| *35S*:CsMYB60-R | GAGCTCTCATGACAAAAGCCAAGAAATCAC(SacI) |
| *35S*:CsbHLH42-F | GGATCCATGGATTCCGACAGCTCGACCA(BamHI) |
| *35S*:CsbHLH42-R | GAGCTCTCAAGTATCAACACAGGGTATG(SacI) |
| *35S*:CsMYC1-F | CCCGGGATGGCTTGCGAACCTGGGTTTC(SmaI) |
| *35S*:CsMYC1-R | CAGCTCTCAGCTCTTGTTGACAACTTTC(SacI) |
| *35S*:CsWD40-F | CCCGGGATGGGTTTTATGTTGACGCTTT(SmaI) |
| *35S*:CsWD40-R | GAGCTCTCACTCATTTCCTGTCTCCTCCT(SacI) |
| *35S*:CsTATA-box-F | GGATCCATGGCAGATCAAGGCTTGGAAG(BamHI) |
| *35S*:CsTATA-box-R | GAGCTCTCATTGCTGGTTTTTCCTGAACTC(SacI) |
| *proCs4CL*-GUS-F | AAGCTTCAATTTCAACCCTAAAACCCTAATAATCTC(HindIII) |
| *proCs4CL*-GUS-R | GGATCCCATAAAATGGGAATGGGATGTTAGTTGT(BamHI) |
| *proCsFLS*-GUS-F | GGATCCAAGCTTTAATCTTCTTGTTCTCTATTTTTC(BamHI) |
| *proCsFLS*-GUS-R | CCCGGGTATCTTTCTTTTAATCTCTTAC(SmaI) |
| *proCsLAR*-GUS-F | GGATCCAATAGTTATTTATCAGAGATCATG(BamHI) |
| *proCsLAR*-GUS-R | CCCGGGTTTCAATAACTTCAAATATCTTC(SmaI) |
| Cs4CL-F | CATCTATGCTGATTCTCCCTC |
| Cs4CL-R | ACAACATCGCCTCCAATC |
| CsCHS-F | ATCTGTCAGTGGAGCGGCCG |
| CsCHS-R | GCTTAGAACCTCTCGAGTTGCTC |
| CsF3H-F | CTTGGGGCTAAAGCGCCATACTG |
| CsF3H-R | GATACATCTCAGAGAAAGTG |
| CsGST-F | CAAGTCCCTGCGTTCCAAGACG |
| CsGST-R | CCTTCCAAGATTCCCTGCCGGAG |
| CsF3'H-F | ATGACATGTTTGATGGTGTTTGG |
| CsF3'H-R | CGTAGGAAGTTTACTTACAAGG |
| CsPAL-F | GTGCTGAGCAACACAATCAAG |
| CsPAL-R | TAGAACTTCCGCTTTCATATGC |
| CsFLS-F | CTTGTGCCGAATGAAGTTC |
| CsFLS-R | CTTACAATATGCATATTCACTG |
| CsLAR-F | CAATATTTAACTTGGAGGAAG |
| CsLAR-R | CGAGAGAGATACTGATCGAC |
| CsCHI-F | CTCCAGTGGAGAAGGTTATAAG |
| CsCHI-R | CAACCACATTTCCATTCTC |
| CsDFR-F | GTAATGAAACAGAGTACGG |
| CsDFR-R | GAGATAACCCTTCTCTTTGC |
| *ProCsFLS*-F | CCCGGGAAGCTTTAATCTTCTTGTTCTC(SmaI) |
| *ProCsFLS*-R | GAGCTCTATCTTTCTTTTAATCTCTTAC(SacI) |
| *ProCs4CL*-F | GAGCTCCAATTTCAACCCTA(SacI) |
| *ProCs4CL*-R | ACGCGTCATAAAATGGGAATGGG(Mlul) |
| *ProCsLAR*-F | CCCGGGAATAGTTATTTATCAGAGATCATG(SmaI) |
| *ProCsLAR*-R | GAGCTCTTTCAATAACTTCAAATATCTTC(SacI) |
| *ProCsCHS*-F | CCCGGGACTCCACCAAAAACTCACAG(SmaI) |
| *ProCsCHS*-R | GAGCTCTGATATGGAAAGAGTTTTTATT(SacI) |
| *ProCsDFR*-F | GAGCTCCACGTGTATATTCCTGAAAG(SacI) |
| *ProCsDFR*-R | ACGCGTCATTTCTCTCGATTTCTACTC(Mlul) |
| probe-CsFLS-F | TTCTTGTGTGTGTTTGTCAGTTGAGTATAAGTTTTGATAT |
| probe-CsFLS-R | ATATCAAAACTTATACTCAACTGACAAACACACACAAGAA |
| probe-muCsFLS-F | TTCTTGTGTGTGTTTGTAAGTTCAGTATAAGTTTTGATAT |
| probe-muCsFLS-R | ATATCAAAACTTATACTGAACTTACAAACACACACAAGAA |
| probe-CsLAR-F | GCAGTCCAAAAAATTCAAC*CCAC*TAACTTTATACCATTAC |
| probe-CsLAR-R | GTAATGGTATAAAGTTAGTGGGTTGAATTTTTTGGACTGC |
| probe-muCsLAR-F | GCAGTCCAAAAAATTCAACG*CAG*TAACTTTATACCATTAC |
| probe-muCsLAR-R | GTAATGGTATAAAGTTACTGCGTTGAATTTTTTGGACTGC |
| probe-CsLAR-2F | GTTAGTGAATGTAAAATTGGTTGTTGATCATTAGCTTAAC |
| probe-CsLAR-2R | GTTAAGCTAATGATCAACAACCAATTTTACATTCACTAAC |
| probe-muCsLAR-2F | GTTAGTGAATGTAAAATAGGTAGTTGATCATTAGCTTAAC |
| probe-muCsLAR-2R | GTTAAGCTAATGATCAACTACCTATTTTACATTCACTAAC |
| CsaV3_3G020110-F | GGCCATTACGGCCATGGCTACGGCTACTCTG(Sfil) |
| CsaV3_3G020110-R | GGCCGAGGCGGCCTCATTTCCAGTTGTTGGCAAC(Sfil) |
| CsaV3_6G039130-F | GGCCATTACGGCCATGGCGGCCATCAATCGG(Sfil) |
| CsaV3_6G039130-R | GGCCGAGGCGGCCCTAAGCTGCTGTTGGAGGC(Sfil) |
| CsaV3_1G031230-F | GGCCATTACGGCCATGGGAATTGACGGTGAC(Sfil) |
| CsaV3_1G031230-R | GGCCGAGGCGGCCTCAAAACGTTGGTACAACGC(Sfil) |
| CsaV3_6G021530-F | GGCCATTACGGCCATGGGGAAAACACGAGG(Sfil) |
| CsaV3_6G021530-R | GGCCGAGGCGGCCTTAAGATCTTGGCTTCTCC(Sfil) |
| CsaV3_2G024370-F | GGCCATTACGGCCATGTCTTCTGGCCGGAAAATC(Sfil) |
| CsaV3_2G024370-R | GGCCGAGGCGGCCTCATTCAAATGCCCATTGG(Sfil) |
| CsaV3_1G046680-F | GGCCATTACGGCCATGTCTCTCACAATCCCCAC(Sfil) |
| CsaV3_1G046680-R | GGCCGAGGCGGCCTTAGACATCTTTGGCAATGAG(Sfil) |
| CsaV3_3G004070-F | GGCCATTACGGCCATGGGTGCAGAAGACACAG(Sfil) |
| CsaV3_3G004070-R | GGCCGAGGCGGCCCTAAGAATTCATAACGGC(Sfil) |
| CsaV3_6G016480-F | GGCCATTACGGCCATGTTCCCTAATCTCCTTCTC(Sfil) |
| CsaV3_6G016480-R | GGCCGAGGCGGCCTCAACTCTTGGAAACTAAGAAAAC(Sfil) |
| GUS-F | ACTTCTGGCCTGGCAGGAGAAAC |
| GUS-R | CTTGCGAGGTCGCAAAATCGGC |
